# Long‐term acclimation to reciprocal light conditions suggests depth‐related selection in the marine foundation species *Posidonia oceanica*

**DOI:** 10.1002/ece3.2731

**Published:** 2017-01-24

**Authors:** Emanuela Dattolo, Lazaro Marín‐Guirao, Juan M. Ruiz, Gabriele Procaccini

**Affiliations:** ^1^ Stazione Zoologica Anton DohrnNapoliItaly; ^2^ Instituto Español de Oceanografía (IEO)San Pedro del PinatarMurciaSpain

**Keywords:** ecological selection, gene expression, light cline, photo‐physiology, reciprocal transplant, seagrasses

## Abstract

Phenotypic differences among populations of the same species reflect selective responses to ecological gradients produced by variations in abiotic and biotic factors. Moreover, they can also originate from genetic differences among populations, due to a reduced gene flow. In this study, we examined the extent of differences in photo‐acclimative traits of *Posidonia oceanica* (L.) *Delile* clones collected above and below the summer thermocline (i.e., −5 and −25 m) in a continuous population extending along the water depth gradient. During a reciprocal light exposure and subsequent recovery in mesocosms, we assessed degree of phenotypic plasticity and local adaptation of plants collected at different depths, by measuring changes in several traits, such as gene expression of target genes, photo‐physiological features, and other fitness‐related traits (i.e., plant morphology, growth, and mortality rates). Samples were also genotyped, using microsatellite markers, in order to evaluate the genetic divergence among plants of the two depths. Measures collected during the study have shown a various degree of phenotypic changes among traits and experimental groups, the amount of phenotypic changes observed was also dependent on the type of light environments considered. Overall plants collected at different depths seem to be able to acclimate to reciprocal light conditions in the experimental time frame, through morphological changes and phenotypic buffering, supported by the plastic regulation of a reduced number of genes. Multivariate analyses indicated that plants cluster better on the base of their depth origin rather than the experimental light conditions applied. The two groups were genetically distinct, but the patterns of phenotypic divergence observed during the experiment support the hypothesis that ecological selection can play a role in the adaptive divergence of *P. oceanica* clones along the depth gradient.

## 1 Introduction

Patterns of genetic and phenotypic variation of plant and animal populations are closely related to the magnitude of environmental heterogeneity in which they live (Conover, Duffy, & Hice, 2009; Endler, 1977; Hice, Duffy, Munch, & Conover, 2012) and are often independent from geographic distances (Richardson, Urban, Bolnick, & Skelly, 2014). Differential response of distinct populations to local environmental features either can fall within the phenotypic plasticity range of the species (Hall et al., 2007; Pfennig et al., 2010; Schlichting, 1986; Thibert‐Plante & Hendry, 2011) or can be due to evolutionary changes with underlying genetic differentiation among populations (Dowdall et al., 2012; Sanford & Kelly, 2011; Savolainen, Lascoux, & Merilä, 2013). When changes in environmental conditions occur, species can shift their distributional range (i.e., Parmesan & Yohe, 2003; Perry, Low, Ellis, & Reynolds, 2005) in order to migrate in more suitable habitats. Alternatively, organisms can compensate environmental fluctuations with phenotypic plasticity (Franks, Sim, & Weis, 2007; Franks, Weber, & Aitken, 2014; Ghalambor, McKay, Carroll, & Reznick, 2007; Gienapp, Teplitsky, Alho, Mills, & Merilä, 2008; Merilä & Hendry, 2014), which can be highlighted at different hierarchy levels of the biological organization, through adjustments of gene expression (i.e., Granados‐Cifuentes, Bellantuono, Ridgway, Hoegh‐Guldberg, & Rodriguez‐Lanetty, 2013; Jeukens, Bittner, Knudsen, & Bernatchez, 2009; Larsen, Nielsen, Williams, & Loeschcke, 2008; Larsen et al., 2007; Pavey, Collin, Nosil, & Rogers, 2010; Swindell, Huebner, & Weber, 2007), developmental features (i.e., Sultan, 2000; West‐Eberhard, 2005), and life‐history traits (Dowdall et al., 2012). Moreover, species can also evolve genetic differentiation among populations (i.e., local adaptation; Kawecki & Ebert, 2004) due to ecological selection (Keller & Seehausen, 2012; Schluter, 2009) and intraspecific competition (García‐Ramos & Huang, 2013). The increase in adaptive divergence also facilitates neutral genetic divergence among populations through the so‐called isolation by adaptation (Nosil, Egan, & Funk, 2008), generating a positive correlation between adaptive phenotypic divergence and neutral genetic differentiation. In case organisms are not able to fulfill adequate strategies to cope with environmental changes, populations may decline and go locally extinct (Chevin, Gallet, Gomulkiewicz, Holt, & Fellous, 2013; Chevin, Lande, & Mace, 2010; Hoffmann & Sgro, 2011; Orr & Unckless, 2008). Thus, the response of organisms to the environment depends on two main evolutionary mechanisms: local adaptation and phenotypic plasticity. Although these processes are not opposite and can coexist, they can produce very different effects at population's level. Through phenotypic plasticity, genotypes are able to fit with a wide range of environmental conditions (within their reaction norm; Pigliucci, 2001), without changes in the genetic makeup of populations. The process of local adaptation, instead, represents a slower response with respect to plasticity but produces changes at the level of the whole population. The two processes interact, as phenotypic plasticity itself is a selective trait (Scheiner & Lyman, 1989). It influences the fitness of single genotypes, affecting also genetic diversity of adapted populations (Agrawal, 2001; Sultan & Bazzaz, 1993).

The incidence of one or the other mechanism depends on the scale (Endler, 1977) and constrains (Callahan, Maughan, & Steiner, 2008; Van Kleunen & Fischer, 2005) of the environmental variations, as well as on the biological and ecological characteristics of the species, as in particular for productive rate and dispersal potential (Baythavong, 2011). Theory predicts that local adaptation is stronger in low‐connected populations (Savolainen et al., 2013) and across strong ecological gradients (Endler, 1977). It can also affect gene flow in close‐by populations, by reducing the survival of immigrants compared to the locally adapted genotypes, due to the mismatch between phenotypes and environments (Edelaar, Siepielski, & Clobert, 2008; Marshall, Monro, Bode, Keough, & Swearer, 2010). Distinguishing between causes and consequences of among‐individual variation for the evolution and persistence of populations (Valladares et al., 2014; Wennersten & Forsman, 2012) will increase our understanding of the ecological dynamics of natural populations and communities, as well as improving strategies for management and conservation of biodiversity.

Although habitat continuity seems to be stronger in the marine than in the terrestrial environment, marine species can display strong genetic differentiation at small spatial scales, due to the fine‐grained variation of abiotic and biotic factors (Cowen & Sponaugle, 2009; Sanford & Kelly, 2011). This also because marine species leave in a multidimensional space, where depth is one of the most important factors in partitioning diversity (Bongaerts et al., 2013; De Carvalho, Chaves, Ormond, Mcginty, & Ferreira, 2012). The light gradient represents one of the main limiting factors for marine photo‐autotrophs, shaping their vertical distribution according to their photo‐acclimation plasticity. Light irradiance decreases exponentially along the water column, and the spectral quality is rapidly altered, due to water absorption and scattering processes (Kirk, 2011). The maintenance of an efficient photosynthetic activity and a positive carbon budget under these wide ranges of light habitats implies several functional adaptations of plant's morphology and physiology.

Seagrasses form high valuable ecosystems (Barbier et al., 2011; Costanza et al., 1997), playing fundamental roles as foundation species, primary producers, and nutrient cyclers worldwide (Larkum & Orth, 2014). Seagrasses spread along the coastline, a highly naturally unstable environment, and are able to cope with strong variation of environmental factors and multiple potential stressors (Hughes, Williams, Duarte, Heck, & Waycott, 2009; Orth et al., 2006; Waycott et al., 2009). Unless the high phenotypic plasticity displayed by some seagrass species (Bricker, Waycott, Calladine, & Zieman, 2011; Sculthorpe, 1967), a general decline of meadows has been recorded, especially in the temperate zones (Short et al., 2011; Telesca et al., 2015), where environmental changes due to anthropogenic impact and global warming are stronger (Reusch & Wood, 2007).

Distinguishing between adaptive selection and phenotypic plasticity in genetically distinct seagrass populations is crucial for the correct evaluation of their resilience and evolutionary potential, as well as for planning efficient conservation strategies. Phenotypic plasticity, in fact, can provide a buffer and assist rapid adaptation in the context of climate change (Nicotra et al., 2010; Reusch, 2013). Local‐scale patterns of genetic diversity are affected by several interconnected factors, among which reproductive and growth strategies are the most important. Seagrasses are able to spread in space by means of clonal reproduction (Hemminga & Duarte, 2000), allowing single genetic individuals (i.e., clones) to persist in time through clonal fragmentation (Arnaud‐Haond et al., 2012; Larkum & Orth, 2014; Reusch, Boström, Stam, & Olsen, 1999). Hence, genetic diversity and population structure are strongly influenced by the persistence of clones, which also regulates flowering and recruitment dynamics (Jahnke, Olsen, & Procaccini, 2015).

The ecosystem engineering *Posidonia oceanica* is a slow‐growing and long‐lived seagrass species, forming extensive mono‐specific meadows from the surface down to 40 m depth (Duarte, 1991), with clones able to persist for hundreds of years (Arnaud‐Haond et al., 2012; Migliaccio, De Martino, Silvestre, & Procaccini, 2005; Procaccini, Orsini, Ruggiero, & Scardi, 2001; Rozenfeld et al., 2007). The depth cline affects the phenology of the species, inducing a delay of about 2 months in the reproductive time of shallow and deep stands of the same meadow (Buia & Mazzella, 1991). This condition can represent an intrinsic barrier to gene flow and can promote the emergence of divergent selection between the two stands. Indeed, a consistent level of genetic differentiation between depths has been recorded in various populations (D'Esposito, Dattolo, Badalamenti, Orsini, & Procaccini, 2012; Migliaccio et al., 2005; Procaccini et al., 2001). Moreover, plants growing at different depths show differences in their response to temperature (Marín‐Guirao, Ruiz, Dattolo, Garcia‐munoz, & Procaccini, 2016) and in many phenotypic characters also affecting meadow structure (Zupo et al., 2006), such as: leaf morphology (Dalla Via et al., 1998), photo‐physiology (Dattolo et al., 2013, 2014; Mazzuca et al., 2013; Pirc, 1986), growth, and respiration (Olesen, Enriquez, Duarte, & Sand‐Jensen, 2002).

Here we present the results of a transplantation experiment in mesocosms, where *P. oceanica* plants collected at two depths (−5 and −25 m) within the same continuous population were exposed to reciprocal light regimes. We applied a combined approach to test the magnitude of photo‐acclimation plasticity and the degree of adaptive differentiation of the shallow and deep individuals. During reciprocal light exposure and subsequent recovery, we measured gene expression of several target genes, photo‐physiological features, and plant morphological and fitness traits. Hence, we interpreted phenotypic patterns of photo‐acclimation in light of the population genetic diversity pattern obtained using neutral microsatellite markers. Moreover, we attempted to identify candidate genes and processes putatively involved in the adaptive differentiation at different light environments.

## 2 Materials and Methods

### 2.1 Study area, plant sampling, and experimental design

Plant material was collected in the last week of August 2013 in three shallow (−5 m) and three deep (−25 m) sampling points selected along a large (ca. 20 km) *P. oceanica* meadow (southeast Spain; Figure S1). In each sampling point, eight plant fragments, consisting of an apical portion of a horizontal rhizome bearing a large number of vertical shoots (hereinafter, ramets), were collected by scuba diving and rapidly transported to the laboratory (<2 hr) to be transplanted in individual pots in a mesocosm facility.

**Figure 1:**
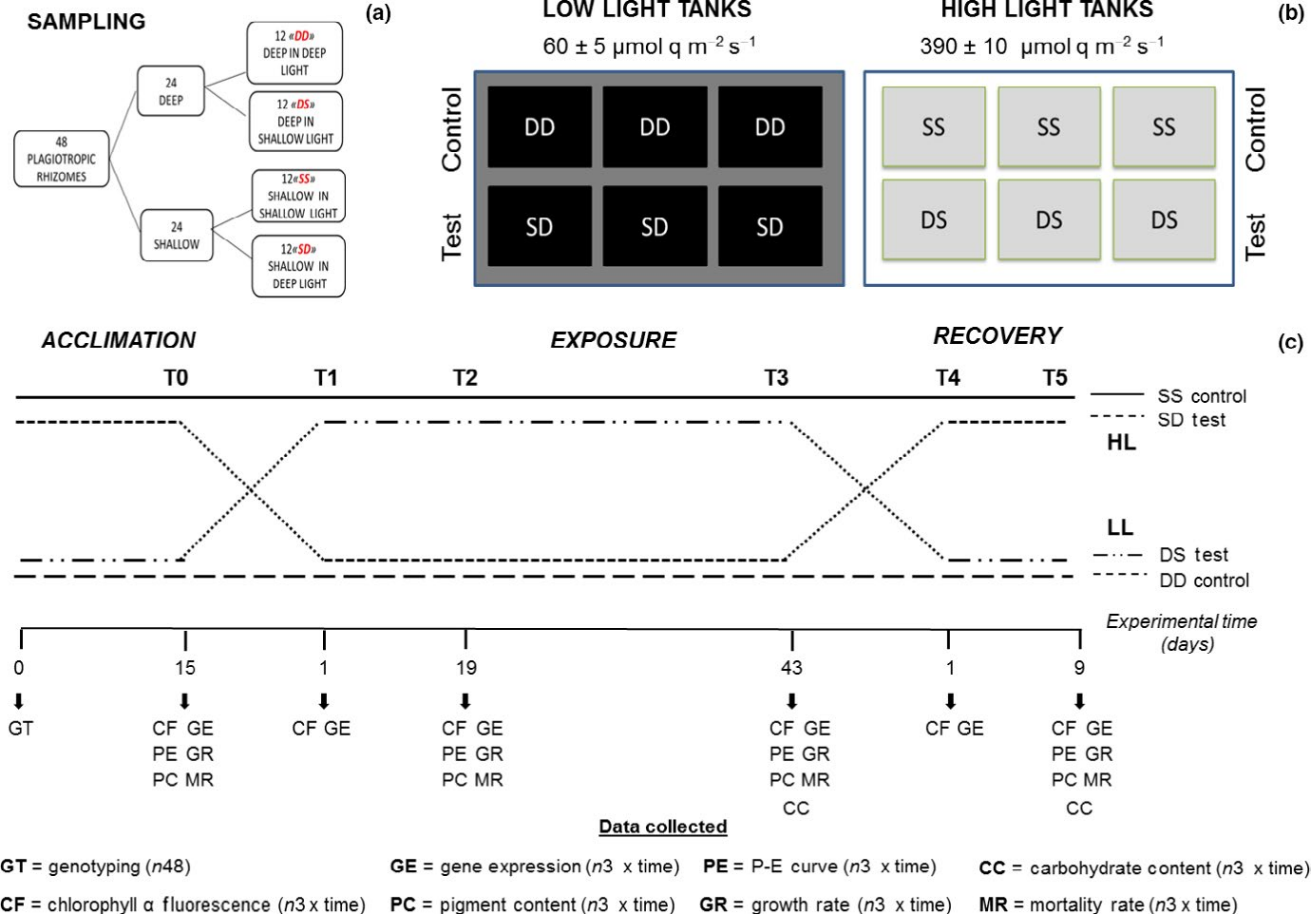
Experimental scheme. (a) Diagram of rhizomes distribution among the four experimental groups: *control*: SS shallow plants in shallow light levels; DD deep plants in deep light levels; *test*: SD shallow plants in deep light levels; DS deep plants in shallow light levels. (b) Schematic representation of the experimental systems. For each stand, 12 plants were used as “control” and 12 as “experimental” groups. Each group is composed of three experimental tanks. SS refers for the tanks containing *P. oceanica* rhizome collected in the shallow stand (−5 m) and maintained in high light. DD refers to the tanks containing *P. oceanica* rhizomes collected in the deep stand (−25 m) and maintained in low light. SD refers for the tanks containing *P. oceanica* rhizomes collected in the shallow stand (−5 m) and exposed to low light. DS refers for the tanks containing *P. oceanica* rhizomes collected in the deep stand (−25 m) and exposed to high light. (c) Experimental time course: acclimation: 2 weeks. At this time plants were genotyped and divided into each experimental and control groups according to their genetic features; reciprocal exposition: 6 weeks; recovery phase: 9 days. Sampling time points: T0 → end of acclimation; T1 → 24 hr after the start of the exposure phase; T2 → 19 days of exposure; T3 → 43 days of exposure; T4 → 24 hr after the start of the recovery phase; T5 → 9 days of recovery. (c) List of data collected along the experiment with the number of biological replicates analyzed at each sampling point for each of the control and experimental tanks. GT, genotyping (all collected genotypes: 48); CF, chlorophyll *a* fluorescence (4); GE, gene expression (1); PC, pigment content (2); PE, P–E curves (2); MR, mortality rate (2); GR, growth rate (4); CC, carbohydrates content (4)

The mesocosm consisted of 12 independent tanks (see Marín‐Guirao, Sandoval‐Gil, Ruíz, & Sánchez‐Lizaso, 2011 for a complete description of the system), each receiving four different ramets according to their bathymetric origin (Figure 1a, b), shallow and deep ramets containing 36 ± 2 and 26 ± 1.3 (mean ± SE) connected shoots, respectively. Light was independently applied in each tank with 400 W metal halide lamps fitted with light neutral diffusing covers. Plants were acclimated at a temperature of 19.5°C during 2 weeks under their respective natural light levels: 390 ± 10 and 60 ± 5 μmol q m^−2^ s^−1^ above the canopy of shallow and deep plants, respectively, both with a 10‐hr:14‐hr light–dark photoperiod (Figure 1b). These irradiances corresponded to a daily integrated photosynthetic photon flux density of 14.04 μmol q m^−2^ day^−1^ for the shallow light condition and of 2.16 μmol q m^−2^ day^−1^ for the deep condition according to mean natural values for the whole period in which the experiment was conducted (i.e., from September to November).

After the acclimatization period, the irradiance in half of the tanks with shallow and deep plants was progressively changed during 3 days until reaching their reciprocal light levels (Figure 1c). This means that shallow plants experienced an 85% of light reduction and deep plants a 6.5‐fold increase in their original light levels. Finally, after 43 days of exposure to their reciprocal light levels, irradiance was again progressively changed to their original levels and maintained during 9 days for recovery. Four experimental treatments were considered: shallow plants in shallow light levels (SS) and deep plants in deep light levels (DD), as control groups; shallow plants in deep light levels (SD) and deep plants in shallow light levels (DS), as test groups (Figure 1a).

### 2.2 Data collections

Plant response was determined at six time points (Figure 1c), namely at the end of the acclimation period (T0), during the exposure period at day 1 (T1, first day of the exposure period), day 19 (T2), and day 43 (T3, last day of the exposure period), and during the recovery period at day 1 (T4, first day of the recovery period) and day 9 (T5, last day of the recovery period). Measures of each plant variable were performed on one to four ramets within each tank and the averaged value used as individual replicate (*n* = 3). A description of the sampling time points and number of ramets employed for each plant variable is summarized in Figure 1c. In brief, the functioning of the photosynthetic apparatus (chlorophyll *a* fluorescence) was determined at all sampling points for all four ramets located in each tank. Photosynthetic and respiratory responses (P–E curves) and leaf pigment content were determined at T0, T2, T3, and T5, for two ramets per tank (Figure 1c). Gene expression was determined at all sampling time points in one ramet per tank. Analyses of plant morphology, leaf growth, carbohydrates content in leaves, and rhizomes were performed in each ramet, at the end of both the exposure (T3) and recovery (T5) periods. Changes in shoot number per ramet were measured in each ramet at T0, T2, T3, and T5 (Figure 1c).

### 2.3 Genotyping and population structure analysis

Genotyping was carried out at the beginning of the experiment, in order to distribute an equal number of genotypes for each test and control groups. A 2‐cm‐long leaf portion was collected for each ramet, cleaned from epiphytes, and stored in silica gel. Genomic DNA was extracted as described in Tomasello et al. (2009). Individual multilocus genotypes were assessed by a total of 29 microsatellites (SSR) (Molecular Ecology Resources Primer Development Consortium et al., 2013). All SSR were combined in four PCR multiplex reactions (for protocol details, see Molecular Ecology Resources Primer Development Consortium et al., 2013). Clonal diversity was estimated using the software Gimlet (Valière, 2002). Using the software GenAlEx v6.5 (Peakall & Smouse, 2012), the diversity indices (i.e., heterozygosity and allelic richness) and the percentage of polymorphic loci were calculated for each tanks (i.e., DD, DS, SS, SD) separately. With the same software (GenAlEx 6), an analysis of molecular variance (AMOVA) was carried out in order to evaluate differentiation between shallow and deep stand, while a principal coordinates analysis (PCoA) calculated on pairwise genetic distance for SSR data sets was performed to evaluate the differentiation among the six sampling points.

### 2.4 Gene expression: RNA extraction and RT‐qPCR analyses

A 4‐cm leaf segment of each ramet placed in the mesocosm was collected, cleaned, and dipped into RNA‐later solution for the analysis of gene expression, for a total of 48 samples for each time point. Three individuals for each treatment were chosen for gene expression analysis among those selected for photo‐physiology. As the same ramet was sampled in every sampling point, the level of expression of TGR (target genes) for each individual was followed along the course of the experiment. Leaf material was always collected between 15 and 16 p.m. to account for the effects of the circadian fluctuation in gene expression.

Total RNA was extracted as described in Mazzuca et al. (2013). RNA quantity and purity were assessed by Nanodrop (ND‐1000 UV–Vis spectrophotometer; NanoDrop Technologies) and 1% agarose gel electrophoresis. Total RNA (500 ng) was reverse‐transcribed in complementary DNA (cDNA) with the iScript^™^ cDNA Synthesis Kit (Bio‐Rad) using the GeneAmp PCR System 9700 (Perkin Elmer).

Transcriptional resources available for *P. oceanica* (data sources: DrZompo database, Wissler et al. 2009; Illumina RNA‐sequencing BioProject ID: PRJNA315106 at DDBJ/ENA/GenBank, under the accession GEMD01000000.) were scored to select suitable targets belonging to the topics: “Photosynthesis,” “Photoprotection,” “Carbon metabolism,” “Photoreceptors,” and “Photoperiod.” Among the selected genes (Table S1), fourteen ones were already investigated in other studies (i.e., Dattolo et al., 2014; Lauritano et al., 2015), while the other sixteen were tested for the first time in this work.

All the gene‐specific primers selected for this study were designed using the tool PRIMER3 (Rozen & Skaletsky, 2000) implemented in UGENE v.1.14.0 (Okonechnikov et al., 2012). Primer specificity was obtained aligning sequences of each target with putative homologous using the BLASTX search tool (Remote NCBI BLAST tool UGENE) in a multiple sequence alignment performed against public database. All primers were designed on sequence's regions with a good BLAST score (*e*‐value 1E^−4^ or below). Primers were validated using a pooled cDNA sample; a standard curve was generated using five serial dilutions of pooled cDNA. All cDNA amplicons ranged from 100 to 250 bp in size to ensure similar PCR efficiency. Reverse transcription quantitative polymerase chain reaction (RT‐qPCR) experiments were conducted in triplicate for each sample, to capture intraassay variability. For each primer's pair and for each assay, a no‐template negative control (NTC) was included. PCR conditions were optimized on a GeneAmp PCR System 9700 (Perkin Elmer) (see Serra et al., 2012 for a detailed description). Amplification efficiency (E) for all primer pairs has been calculated from the slopes of standard curves of the threshold cycle (CT) versus cDNA concentration, with the equation *E* = 10^−1/slope^−1. All *E* were ≥90%, and *R* ^2^ was always >.9 (Table S1).

The expression stability of a set of putative reference genes already tested in *P. oceanica* (Serra et al., 2012) and all putative target genes selected for this work (Table S1) was analyzed together in both light treatments. Stability values were validated with the three softwares GeNorm, Normfinder, and BestKeeper as described in Serra et al. (2012). According to stability analysis, two reference genes, namely ubiquitin‐conjugating enzyme (NTUBC) and Gigantea (GI) (i.e., REF in Table S1), were used to normalize gene expression data. The putative reference gene glyceraldehyde 3‐phosphate dehydrogenase (GAPDH) was included in the analysis, for a total of 28 target genes (i.e., TGR in Table S1). Target genes related to “Photoprotection” were tested only in high light condition (i.e., in DS and SS samples).

In order to follow the effect of light treatment along the experimental time points, we firstly assessed the relative expression level of target genes normalized against the reference genes (ΔCT values) in the four tester/control groups. Subsequently, results were tested for significance considering the variation of gene expression among the four groups (SD, DS, SS, and DD) during the two reciprocal exposure and recovery periods (RM‐ANOVA, see the following statistical data analyses section).

In order to monitor the time course of acclimatization and recovery processes of the two test groups (i.e., SD and DS), we assessed the difference in mRNA transcript levels among each couple of tester/control using REST 2002 (ΔΔCT, relative expression software Tool, ver 2.0.13; Pfaffl, Horgan, & Dempfle, 2002). Two kinds of comparison were assessed: (i) “Home versus Away,” to evaluate the relative expression ratio between test groups against the own “population” controls (SD vs. SS and DS vs. DD) and (ii) “Native versus Foreign,” to evaluate the relative expression ratio between test groups against the own “environmental” control (SD vs. DD and DS vs. SS).

### 2.5 Photo‐physiology

Chlorophyll a fluorescence measurements were performed with a diving‐PAM fluorometer (Walz, Germany) as described in Marín‐Guirao et al. (2013). Measurements were initially taken on whole night dark‐adapted leaves to determine the maximum quantum yield of PSII (Fv/Fm). Subsequently, rapid light curves (RLC) were generated on the same leaf area after 4 h of illumination in the mesocosm to determine the effective quantum yield of PSII (DF/Fm’) and the relative electron transport rate (rETR).

Photosynthetic and respiratory rates were determined following previously described methods (Marín‐Guirao et al., 2011). Leaf segments of 2 cm^2^ from the middle part of the first mature leaf were incubated in a DW3 incubation chamber housing a Clark type O_2_ electrode (Hansatech, UK) connected to a controlled‐temperature circulating bath. In each incubation, leaf segments were maintained at the same temperature as the aquaria (19.5°C) and exposed to increasing light intensities (0, 25, 100, 300 μmol photons m^−2^ s^−1^). After the final light exposure, leaf segments were exposed to darkness to determine dark respiration rates (Rd). From each incubation, gross photosynthetic rates (gross‐Pmax) were also determined and expressed as μmol O_2_ g^−1^ h^−1^.

Leaf pigment content was determined in the same leaf segments employed for the analysis of photosynthesis and respiration. Pigments were extracted from 1 cm^2^ leaf segments in buffered acetone, and the absorbance of the extracts was spectrophotometrically read to calculate chlorophyll a, b and total carotenoids (Lichtenthaler & Wellburn, 1983) and expressed per biomass (μg g^−1 ^F.W.).

### 2.6 Plant morphology and fitness traits

The content of carbohydrates was analyzed in leaf and rhizome tissues. Each leaf sample was composed by the healthy leaves of three vertical shoots from the same ramet, while the first 2 cm of their rhizome apex composed the rhizome sample. The content of nonstructural carbohydrates (soluble fraction and starch) was determined using the anthrone assay protocol according to methods detailed in Marín‐Guirao et al. (2013). At the beginning of both the reciprocal light exposure and recovery periods, six *P. oceanica* shoots were marked in each ramet to determine leaf growth rates and shoot morphology. Three marked shoots were subsequently harvested from each ramet to determine mean values of shoot leaf growth at the end of both periods (T3 and T5). The number of leaves per shoot, shoot size, production of new leaves per shoot (i.e., leaves without mark), and percentage of the senescent leaf area were also measured on harvested shoots. All shoots in each ramet were counted at the end of the acclimation period (T0) and at the end of both the exposure (T3) and recovery (T5) periods, to calculate the percentage of net change in shoot number.

### 2.7 Statistical analyses

An analysis of similarity (ANOSIM) was performed to assess the differences in gene expression patterns among control and test groups at the different time points using the PRIMER (Carr, 1996) software package V6 (Clarke & Gorley, 2001). In addition, a SIMPER analysis was carried out to identify genes most responsible for differences between groups. For both analyses, Bray–Curtis matrix was obtained for each group from the relative gene expression values (−ΔCT values).

One‐way repeated‐measures ANOVA was applied to assess the statistical significance of the effects of treatment on gene expression and photo‐physiological parameters (i.e., chlorophyll a fluorescence, P–E curves, and pigment content) over the experimental periods. Data were first checked for homogeneity of variance using Levene's test and transformed when necessary. If the assumption was still not met, the *p* value was set to .01 to minimize the risk of a type I error (Underwood, 1997). Both exposure and recovery periods were separately analyzed, with the final day of exposure (T3) forming both the last sampling of the reciprocal light treatment and the first day of the recovery. Treatment (four levels: DD, DS, SS, and SD) was the between‐subject factor and time points the repeated‐measures (within‐subject) factor. RM‐ANOVAs were carried out according to the procedures described in Quinn and Keough (2002). Before running the analyses, the variance‐covariance matrices were tested for sphericity using Mauchly's test, and if the assumption was not met (*p* < .05), the Greenhouse–Geisser (G–G) epsilon adjustment was applied to the degrees of freedom. The treatments and time effects identified, using RM‐ANOVA, were interpreted using Newman–Keuls post hoc analysis. One‐way ANOVA was used for those parameters derived from P–E curves and pigment analysis in the recovery period (T5).

Similarly, for data collected only at the end of the exposure (T3) and recovery (T5) periods (i.e., plant morphology and fitness traits), one‐way ANOVA was performed for each period with treatment as a fixed effect. To identify similar patterns in photo‐physiological responses and gene expression among control and test groups, multidimensional ordinations based on PCA were performed on each data set with the software PAST3 (Hammer, Harper, & Ryan, 2001). For PCA of gene responses, the expression value of target genes (‐ΔCT values) was employed in the analysis. To investigate biological significance of differential expression observed among our multiple comparisons, a combined approach of fold‐change cutoff and statistical significance along the time course was applied. To assess the degree of plasticity in expression of target genes with environmental or population effects, we considered post hoc Tukey's test, to test for differences in expression level among the four groups and the relative expression values. Target genes were considered plastic (i.e., with environmental effect) if mean expression levels differed in comparisons between groups of the same population (i.e., depth), but were not significantly different between groups of different depths exposed to the common light treatment.

Conversely, targets genes were considered less plastic (i.e., with population effect) if mean expression levels differed in comparisons between groups exposed to the same light treatment, but were not significantly different between groups of the same native depth although exposed to different light treatment.

To evaluate the acclimation rate along the experimental time course, pairwise comparison of each test groups against the own environment control (“Home vs. Away,” i.e., SS vs. SD and DD vs. DS and “Native vs. Foreign,” i.e., SS vs. DS and DD vs. SD) at each time point was considered significant only if expression ratio was higher than ±2 log2 fold (∆∆CT data).

## 3 Results

### 3.1 Irradiance levels in experimental treatments

Light reaching the leaf canopy in aquaria was (mean ± SE) 384 ± 7 and 67 ± 1 μmol q m^−2^ s^−1^ for shallow and deep light treatments, respectively. Photo‐physiological parameters were obtained within the canopy, and the leaf fragments for the analysis of gene expression were taken from approximately 20–25 cm above the leaf sheath, where irradiances were 184 ± 4 and 40 ± 1 μmol q m^−2^ s^−1^, for shallow and deep light treatments, respectively. This reflects a higher light reduction in shallow experimental canopies (51%) than in deep ones (40%) as a consequence of the natural differences in canopy structure (i.e., shoot density) among shallow and deep ramets.

### 3.2 Genotyping of transplanted shoots and population structure

**Figure 2:**
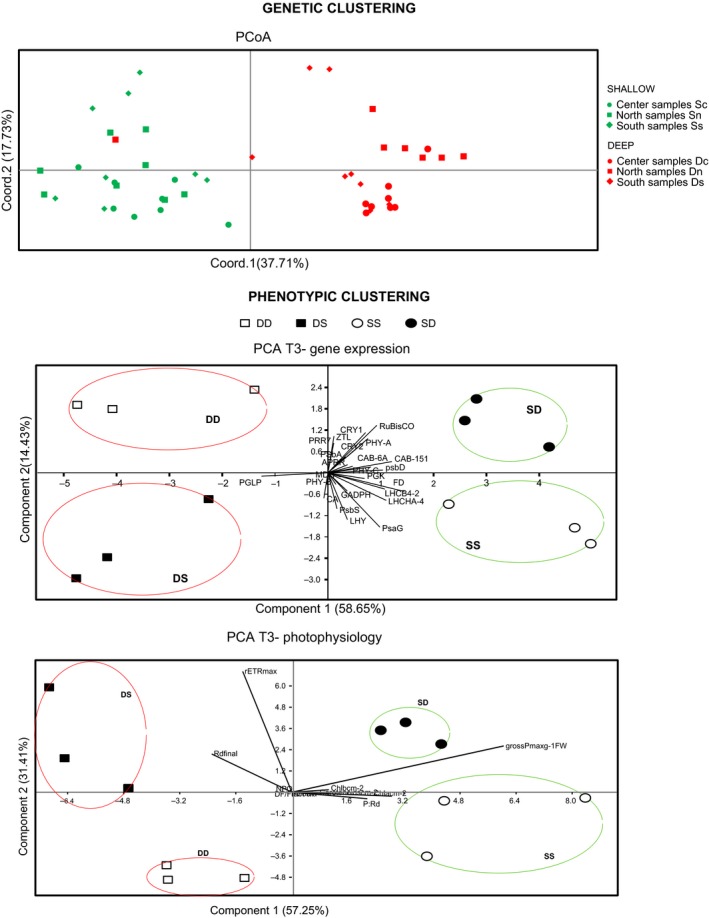
Genetic and phenotypic clustering. (a) PCoA plots of the first two axes based on SSR distance matrices. Percentage of explained variance of each axis is given in parentheses. Individual samples are represented by a symbol according to its sampling station. (b) Phenotypic cluster of gene expression data. Global pattern of transcriptional responses on the four experimental groups at the end of exposure (T3).PC1 and PC2 explain the 58.65% and 14.43% of the variation, respectively. (c) Phenotypic cluster of photo‐physiological data. Global pattern of photo‐physiological variables on the four experimental groups at the end of exposure (T3). PC1 and PC2 explain the 57.25% and 31.41% of the variation, respectively

Almost all collected ramets were distinct genotypes (46 over 48), with only two replicated genotypes among the deep samples. The main genetic diversity indexes were very similar among the experimental groups (DD, DS, SS, and SD; Table S2a), although most of the genetic parameter assessed were slightly higher in shallow plants. The percent of polymorphic loci ranged from 68.97 in DD to 82.76 in SS, the number of alleles ranged from 10.59 in SD to 11.69 in DS, the observed heterozygosity ranged from 0.417 in DS to 0.483 in SD, and the expected heterozygosity from 0.284 in DD to 0.398 in SD (Table S2a). Although the analysis of molecular variance showed that depth explains only 12% of the total variation, the value of population differentiation was low but significant (*F*_st_ = 0.141, *p* = .001; Table S2c). Patterns of genetic subdivision revealed by the PCoA showed strong support for two main groups that correspond to the two depths (Figure 2a), although one genotype from the deep stand was most likely assigned to the shallow meadow (Figure 2a).

### 3.3 Photo‐acclimatization to contrasting light environments: gene expression of target genes

The relative expression (ΔCT) of each target gene assessed in the four groups across all time points is reported in Supplementary material (ΔCT values, Figure S2a‐c). In the same figure, the statistical significance of each pairwise comparison is also reported.

The complete RT‐qPCR results of each comparison (“Home vs. Away” and “Native vs. Foreign”) during the time course of the experiment are reported in Table S3a‐d (ΔΔCT values). In Figure S3a, b only the relative expression profiles of each test group (i.e., DS and SD) against their environmental control during exposure and recovery are reported.

According to Tukey's post hoc test, expression levels of 11 target genes exhibited significant population or environmental treatment effects (Figure S2a‐c) along the experiment. In particular, target genes belonging to “Photosynthesis” (Figure S2a), such as light‐harvesting proteins and structural components of photosystems (PSAG and psbD), displayed several significant differences in their expression values (ΔCT) both between control groups (SS, DD) and in pairwise comparisons of tester and control groups, especially during the acclimatization phase. Targets belonging to “Carbon metabolism” showed statistically significant differences both between population and treatment controls (i.e., RbcS in T2, T3 and T5, PGLP in T3; Figure 2Sb). Expression level of “Photoprotective/Antioxidants” genes was similar in the four groups, while among “Circadian” and “Photoreceptors,” most targets (APRR, PHYA, PHYC, CRY1) revealed significant differences in several pairwise comparisons (Figure S2c).

Moreover, comparisons “Home versus Away” were made to identify genes which exhibited a plastic pattern of gene expression between test and control groups of the same population and thus are the most involved in the acclimation processes (see, e.g., Swindell et al., 2007). In the “Home versus Away” comparisons (Table S3a, c), shallow plants (i.e., SS vs. SD) during the low‐light acclimation exhibited plastic regulation in five target genes (i.e., LHCHA‐4, PSBS, psbA, PHYC, and ZTL; Table S3a). For deep plants under high light (i.e., DD vs. DS), eight genes (LHCHA‐4, LHCB4‐2, psbD, PSBS, CRY1, CRY2, APRR, and PGLP; Table S3c) were identified as the more plastic during light acclimation.

During the recovery (T4–T5), only few target genes were differently regulated (i.e., five and six genes in shallow and deep plants, respectively; Table S3a, c). psbD is the only responsive gene in T5, where it is up‐regulated (3.10 log2 fold‐change) in shallow samples exposed to low light (SD) and down‐regulated (−4.59 log2 fold‐change) in deep samples exposed to high light (DS).

In the “Foreign versus Native” comparisons, 11 (CAB‐151, PSAG, psbD, psbA, FD, PGK, MDH, PHYC, ZTL, LHY, and APRR) and six target genes (LHCB4‐2, LHCHA‐4, CAB‐151, CAB‐6A, FD, and RbcS) were differentially regulated along the reciprocal light exposure in shallow (SD vs. DD) and deep (DS vs. SS) plants, respectively (Table S3b, d). Looking more in detail to each comparison along the experimental time course, it is noteworthy that both test groups (DS and SD) displayed a rapid change of gene expression after 1 day of light change (T1). Moreover, most genes showed an opposite regulation pattern between high and low light; indeed, those that were up‐regulated in SD were down‐regulated in DS and vice versa (see T1, Figure S3a, b and Table S3b, d). Native shallow plants exposed to deep light intensity (SD) displayed in T1 a general up‐regulation of transcripts belonging to “Photosynthesis,” in comparison with the deep control plants (DD). Several other genes related to “Photoreception and Photoperiod” also displayed statistical significant fold‐changes (i.e., APRR down‐regulated, PHYA and CRY1 up‐regulated; Table S3b, Figure S3a). Almost all target genes were up‐regulated in T2 and T3 (Table S3). LHCB4‐2, CAB‐151, psbD, PHYC, and CRY1 were statistically significant in T2 and PHYA in T3. PGLP, however, was the only significantly down‐regulated in T3 (Figure S3a, Table S3b). After 1 day of recovery, in T4, mRNA expression level of SD was comparable with the own environmental control (SS) suggesting a fast recovery. Indeed, at the end of recovery (T5), only one gene showed statistically significant differences between test and control (i.e., PHYC) while only psbD was up‐regulated (Figure S3a).

Deep plants (DS) exposed for 1 day (T1) to high light intensity and compared with native high‐light plants (SS) showed a notable down‐regulation of many transcripts involved in “Photosynthesis” (Figure S2b; Table S3c, d) as, for instance, psbD, encoding for the protein D2, and the photoreceptor CRY1, which were significantly down‐regulated. At the end of the exposure period (T3), almost all target genes displayed a down‐regulation in deep test plants, the four genes LHCB4‐2, LHCA‐4, PHYA, and CRY1 being statistically significant. Only PGLP was slightly up‐regulated. At the end of the recovery period (T5), most of the genes in DS plants displayed just minor differences in comparison with plants growing in their natural low light condition (DD). CAB‐151, RbcS, and APRR were significantly different from the control (Figure S2a).

The ANOSIM distinguished four significantly different groups (SD, DS, SS, and DD) at each time point according to their overall expression profiles (one‐way ANOSIM; Table S4). SIMPER analysis following the ANOSIM revealed that genes belonging to “Photosynthesis,” “Photoreception,” and “Photoperiod” were main responsible for these differences, with a minor contribution of those genes related to “Carbon metabolism” (% SIMPER results; Table S4).

The PCA performed at the end of the reciprocal light exposure (T3) showed that the four treatments clearly segregate in the four panels of the plot (Figure 2a). Plants clustered according to their native depth along the first axis, which explained the 57.69% of the total variance. The second axis explaining 15.46% of the variance separated plants according to the light treatment (high‐light plants were on the positive side of the axis and low‐light plants on the negative one) (Figure 2a). Plants from the deepest stand were positively correlated with PGLP, while plants from the shallow ones displayed the higher positive correlations with FD, RbcS, and two chlorophyll binding proteins (CAB‐151 and LHCA‐4). Genes most positively correlated with PC2 axis were RbcS and CRY1, while PSAG and LHY showed the most negative loadings.

### 3.4 Time course of photo‐physiological variables

Leaf pigment content (chlorophyll *a*,* b* and total carotenoids) of shallow plants was higher than that of deep plants, and the differences were maintained in the controls (SS and DD) all along the experimental period (Figure S4). Shallow plants under deep light (SD) showed a transient pigment reduction in T2 when concentrations were similar to deep plants, but returned to their control levels at the end of the reciprocal exposure period (T3). Pigments of deep plants (DS) did not change during their exposure to high light and showed a generalized reduction (Chl *a*,* b* and total carotenoids), although not significant, just after being returned to their original light (T5) (Table S5).

The maximum quantum yield of PSII (*Fv*/*Fm*) showed no significant modification as a consequence of the light treatments and showed values that were higher than 0.770 in every sampling time and treatment (Figure S5; Table S6). This parameter only responded in DS plants right after the light changes in T1 and T4 when it was reduced and increased, respectively.

The effective photochemical efficiency showed a significant time × treatment effect during the exposure and recovery periods (Table S6). Values were higher in shallow than in deep plants and evidenced significant and rapid responses to light changes. At the end of the reciprocal light exposure (T3), the photochemical efficiency of SD and DS plants equaled that of DD and SS, respectively, although it takes longer to DS than to SD plants. Once these experimental plants were progressively returned to their original light levels, Δ*F*/*Fm*’ values equal those of controls after just 1 day.

The electron transport rate (ETR) showed response similar to the photochemical efficiency one. ETR values were almost double in shallow than in deep plants before the light change in experimental treatments (T0). Nevertheless, just 1 day after growing under their reciprocal light levels, shallow (SD) and deep (DS) plants showed similar ETR values than deep (DD) and shallow (SS) controls, respectively. This response was maintained until the end of the exposure period, after which mean values of SD and DS returned to their respective control levels in just 1 day of recovery.

**Figure 3:**
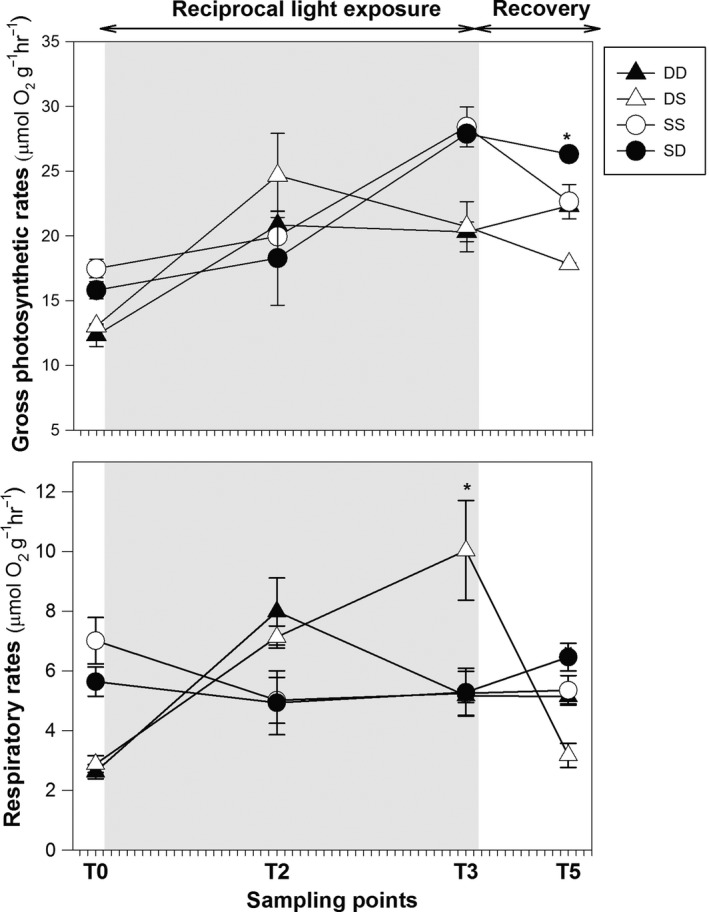
Photosynthesis and photorespiration. Maximum photosynthetic and respiratory rates (μmol O_2_ g^−1^ h^−1^) of experimental plants along the reciprocal light exposure (gray area) and the subsequent recovery periods. Asterisks indicate significant treatment effects (*p ≤ .05)

Photosynthesis and respiration evidenced both a significant treatment x time effect along the reciprocal light exposure (Table S5). Shallow plants under slow light (SD) did not evidence significant changes in their photosynthetic rates neither in their respiratory activity with respect to SS plants (Figure 3). Deep plants under high light (DS) neither modified their gross‐Pmax values but experienced a significant increase in respiratory rates that doubled those of DD plants at the end of the exposure period. At the end of the recovery phase, gross photosynthesis of SD and DS plants was respectively higher and lower than their controls whereas respiration was only affected in DS with values that were significantly lower than in the rest of treatments.

A PCA based on the photo‐physiological data from T3 gave a similar pattern to that obtained with transcriptional data (Figure 2c). The PC1, that explained 57% of the total variance, separated treatments according to the depth origin of plants, with shallow and deep plants plotted, respectively, in the positive and negative sides of the axis and being gross photosynthetic rates the variable with higher positive loadings. PC2 (31% of total variance) instead separated controls from test treatments and was mainly correlated with ETR. Respiration seems to have a very strong implication in segregating DS plants.

### 3.5 Plant morphology and fitness traits

At the end of the reciprocal light exposure, DS plants significantly increased their starch (39%) and soluble sugars (18%) in leaf content in relation to DD controls, whereas in SD plants both energetic components were reduced with respect to SS controls by 27% and 13%, respectively, although not significantly (Table 1). These trends were reversed at the end of the recovery phase when carbohydrates in leaves of DS equal those of DD. Contrarily, when SD plants were returned to their original light conditions at the end of the recovery phase their leaves showed higher carbohydrates concentrations than SS controls. Carbohydrates content in rhizomes of deep plants (DD and DS) was significantly higher than in shallow ones (SS and SD) and did not show significant variations along the exposure and recovery periods.

**Table 1:** Carbohydrates content in leaves and rhizomes

	Treatments				ANOVA results		
	DD	DS	SS	SD	*df*	*F*	*p*
Starch in leaves (%D.W.)							
Exposure (T3)	1.80 (0.18)^b^	2.51 (0.15)^a^	1.14 (0.06)^c^	0.83 (0.08)^c^	3	34.6	***
Recovery (T5)	0.78 (0.01)^b^	0.61 (0.03)^b^	0.64 (0.18)^b^	1.15 (0.04)^a^	3	7.09	*
Soluble sugars in leaves (%D.W.)							
Exposure (T3)	2.99 (0.12)^b^	3.52 (0.08)^a^	1.80 (0.02)^c^	1.57 (0.08)^c^	3	122.7	***
Recovery (T5)	2.48 (0.21)^ab^	2.64 (0.19)^a^	1.76 (0.21)^c^	2.51 (0.16)^ab^	3	4.27	*
Starch in rhizomes (%D.W.)							
Exposure (T3)	5.81 (0.84)^a^	6.79 (0.12)^a^	3.11 (0.40)^b^	1.87 (0.08)^b^	3	9.77	**
Recovery (T5)	7.31 (0.26)^a^	7.48 (0.62)^a^	2.21 (0.10)^b^	1.56 (0.12)^b^	3	84.41	***
Soluble sugars in rhizomes (%D.W.)							
Exposure (T3)	5.24 (0.35)	5.08 (0.63)	2.58 (0.58)	3.19 (0.96)	3	4.0	n.s.
Recovery (T5)	2.51 (0.38)^ab^	3.03 (0.57)^a^	1.09 (0.19)^b^	1.13 (0.33)^b^	3	6.13	*

Carbohydrates (starch and soluble sugars) content in leaves and rhizomes of experimental *P. oceanica* plants at the end of the reciprocal light exposure period (i.e., exposure, T3) and at the end of the recovery period (i.e., recovery, T5). Results of one‐way ANOVA are also shown. Values are means (SE). Different letters indicate significant differences among treatments as indicated in the post hoc analysis (*p ≤ 0.05; **p ≤ 0.001; ***p ≤ 0.005).

Light changes caused morphological modifications in both shallow and deep experimental plants. Shallow plants had a lower number of leaves in T3, when grown under low light conditions (SD) with respect to their natural condition (SS), as well as a 37% lower production of new leaves, a 22% reduction in leaf growth rates, and shorter (16%) and wider leaves (*p* < .05; Table 2). On the contrary, deep plants exposed to increased light levels (DS) produced the same number of new leaves than their controls (DD). Nevertheless, we observed a delay in the shedding of old and lengthy leaves (as reflected by the maximum leaf length and necrotic leaf tissue; Table 2) giving place to plants with a greater number of leaves and thus with a 20% larger size. They also significantly increased (35%) their leaf growth rates.

**Table 2:** Plant morphological characteristics

	Treatments				ANOVA results		
	DD	DS	SS	SD	*df*	*F*	*p*
Shoot size (cm^2^ shoot^−1^)							
Exposure (T3)	100.1 (8.7)^ab^	120.7 (10.8)^a^	78.9 (6.2)^bc^	68.0 (3.6)^c^	3	8.97	**
Recovery (T5)	65.4 (2.8)^a^	92.0 (5.5)^b^	58.1 (2.0)^a^	61.2 (6.9)^a^	3	10.68	**
Leaves per shoot							
Exposure (T3)	4.6 (0.3)^a^	5.3 (0.0)^ab^	5.8 (0.1)^b^	4.8 (0.2)^a^	3	8.10	**
Recovery (T5)	5.0 (0.3)	5.4 (0.2)	5.5 (0.1)	5.0 (0.4)	3	0.87	n.s.
Maximum leaf length (cm)							
Exposure (T3)	49.9 (1.0)^a^	55.1 (2.7)^a^	32.4 (1.0)^b^	27.1 (2.3)^b^	3	48.79	***
Recovery (T5)	33.1 (1.3)^b^	45.8 (4.8)^a^	21.8 (0.3)^c^	21.1 (0.1)^c^	3	21.76	***
Maximum leaf width (cm)							
Exposure (T3)	1.06 (0.01)^a^	1.04 (0.01)^a^	0.94 (0.02)^b^	1.02 (0.02)^a^	3	11.65	**
Recovery (T5)	1.06 (0.01)^a^	1.02 (0.02)^a^	0.91 (0.02)^b^	0.99 (0.03)^a^	3	8.0	**
Senescent leaf surface (cm^2^ shoot^−1^)							
Exposure (T3)	18.8 (1.7)^a^	22.9 (1.9)^a^	11.2 (1.3)^b^	9.9 (2.9)^b^	3	9.43	**
Recovery (T5)	9.9 (0.5)^b^	20.0 (2.5)^c^	4.1 (1.4)^a^	3.9 (0.3)^a^	3	6.98	*
New leaves production (leaves day^−1^)							
Exposure (T3)	0.046 (0.003)^a^	0.058 (0.003)^a^	0.046 (0.003)^a^	0.029 (0.004)^b^	3	14.20	**
Recovery (T5)	0.118 (0.020)^a^	0.066 (0.012)^ab^	0.071 (0.010)^ab^	0.030 (0.006)^c^	3	7.74	**
Leaf growth (cm^2^ shoot^−1^ day^−1^)							
Exposure (T3)	0.62 (0.07)^a^	0.84 (0.01)^b^	1.18 (0.09)^c^	0.93 (0.03)^b^	3	15.52	**
Recovery (T5)	1.71 (0.07)	1.69 (0.04)	1.88 (0.14)	2.01 (0.10)	3	2.67	n.s.
Net shoot change (%)							
Exposure (T3)	102.2 (1.0)	101.8 (0.1)	98.8 (1.6)	96.1 (3.6)	3	2.03	n.s.
Recovery (T5)	100.8 (0.8)	100.9 (1.8)	99.9 (5.0)	91.8 (3.2)	3	1.96	n.s.

Plant morphological characteristics (shoot size, number of leaves per shoot, maximum leaf length and width, senescent leaf surface), growth parameters (leaf growth and the production of new leaves), and net shoot change in experimental treatments at the end of the reciprocal light exposure period (i.e., exposure, T3) and at the end of the recovery period (i.e., recovery, T5). Results of one‐way ANOVA are also shown. Values are means (SE). Different letters indicate significant differences among treatments as indicated in the post hoc analysis (*p ≤ 0.05; **p ≤ 0.001; ***p ≤ 0.005).

At the end of the recovery period, SD plants recovered their leaf growth rates, although their leaves were still significantly wider and the production of new leaves significantly lower. DS plants remained larger than controls because old leaves were still present as indicated by their higher leaf length and percentage in necrotic surface area. These plants, however, recovered their original growth rates equaling those of the controls.

Both shallow and deep plants showed high percentage of survival in T3, and both experimental populations (i.e., SD and DS; Table 2) showed no changes in the number of shoots and at the end of the experiment, with ca. 100% of the original plants still alive and without apparent signs of disturbance. Only two shallow genotypes showed shoot mortality rates up to approximately 20%.

## 4 Discussion

Understanding how phenotypic variation is generated and maintained along environmental clines has important implications for increasing our knowledge on organism's adaptation to variable environments and on the evolutionary processes underlying diversification (Endler, 1977). In the context of global climatic changes, it is also critical to evaluate the population‐level consequences of interindividual genetic and phenotypic variations (Forsman, 2014). Studies with common garden and reciprocal transplants allow to assess the degree of plasticity of traits with a functional correlation with ecological factors and to identify the genetic basis and mechanisms shaping distribution and tolerance limits of populations and species (e.g., Evans & Hofmann, 2012; Robakowski, Li, & Reich, 2012).

Here we studied acclimatization plasticity and plant's fitness in distinct genotypes collected along the depth gradient of the seagrass *Posidonia oceanica* exposed to reciprocal light conditions in a common garden experiment. *P. oceanica* is a slow‐growing plant, with sporadic sexual reproduction, which precludes the possibility to apply a classical experimental design using second‐generation plants. Nevertheless, our experimental approach allowed to estimate the population‐level plasticity in photosynthetic acclimation and to identify fixed traits potentially implied in the ecological differentiation of genotypes growing at different depths.

Measures collected during this study showed a degree of phenotypic changes among traits and experimental groups, which also differed in the two light environments considered. Overall plants collected at different depths seem to be able to acclimate to reciprocal light conditions in the experimental time frame, through morphological changes and phenotypic buffering, supported by the plastic regulation of a reduced number of genes. However, shallow plants in low light conditions show lower potential for long‐term acclimation. Indeed, multivariate analyses of both gene expression and physiological data indicated that photo‐phenotypes of *P. oceanica* tester plants correlated more with the plants original depth distribution rather than the light environment to which each group was exposed and patterns of genetic diversity displayed a clear subdivision of the samples (with the exclusion of two) in two main clusters corresponding to the two depths. Below we discuss the results in the context of photo‐physiological buffering capacity of plants against light changes, identifying more flexible or fixed traits and the range of variation among populations and environments. We also discussed the putative causes and processes driving phenotypic and genotypic cline variations of *P. oceanica* along the depth gradient.

In natural conditions, *P. oceanica* meadows progressively reduce their canopy complexity (e.g., shoot density, canopy height) with increasing depth, regulating the light conditions within the canopy (Dalla Via et al., 1998; Marín‐Guirao, Ruiz, & Sandoval‐Gil, 2015). These structural changes are considered the main adaptive mechanisms to offset depth‐related light reductions (Collier, Lavery, Ralph, & Masini, 2008; Olesen et al., 2002; Ralph, Durako, Enriquez, Collier, & Doblin, 2007). In our analysis, *P. oceanica* plants under reciprocal light levels showed notable morphological plasticity, with distinct morphological changes that appear as strategies for modifying the light environment within the canopy (Enríquez, 2005). Indeed, at the end of the exposure period, shallow plants exposed to low light presented shorter and wider leaves and lower number of leaves per shoot, a strategy to maximize light exposure of photosynthetic tissues and minimize respiratory demand (Ralph et al., 2007). Shallow plants in low light also showed lower growth rate, a feature that has been proposed as an indicator of light reduction in seagrasses, as it responds early and reflects sublethal changes at the plant scale (McMahon, Collier, & Lavery, 2013). On the other hand, deep plants under high light conditions reduced the shedding of old summer leaves. These long necrotic leaves shade and protect the young and mature photosynthetic ones, regulating the light environment to which they are exposed.

Photo‐physiological responses of tester groups reflected different plasticity among clones from the two depths. Only shallow plants showed a rapid modulation (i.e., down‐expression) in the level of expression of transcripts encoding for light‐harvesting components (such as CAB‐151 or LHCs) immediately after beginning the light treatment (T1). Accordingly, the leaf pigment content showed a generalized reduction after a few weeks (T2). The limited variation in gene expression in the long term, however, paralleled the small changes in pigments, not allowing test plants to achieve a pigment composition similar to the controls (T3).

Plants from both depths modified their ability to process harvested light and showed contrasting responses in the expression of transcripts encoding for structural components of the photosystems (PSAG, psbD), which were down‐regulated in shallow plants and overexpressed in deep ones, immediately after the light change. This fast response, that is maintained in the long term, is likely reflecting the activation of molecular mechanisms that promoted the rapid photochemical adjustment observed (Demmig‐Adams, Adams, & Matoo, 2006; Niyogi, Li, Rosenberg, & Jung, 2005). The photochemical efficiency of shallow and deep plants changed along the experiment, matching values of deep (DD) and shallow (SS) controls, respectively, and without evidence of photo‐damage accumulation. The ability to move electrons along the electron transport chain increased in deep (DS) transplants and decreased in shallow (SD) ones, although their photosynthetic capacity (O_2_ production) remained unmodified along the light exposure, evidencing the lack of long‐term acclimation in photosynthetic carbon fixation to imposed light changes. The inconsistency between the two photosynthetic parameters (i.e., photosynthetic capacity and photosynthetic efficiency) might be related to changes in leaf absorptance, which can be promoted by changes in leaf morphology (Enríquez, 2005). Alternatively, it can be due to the potential activation/deactivation under stressful conditions of alternative electron transport pathways (e.g., photorespiration, water–water cycle, cyclic electron transport (Niyogi, 1999, 2000).

The key enzyme in the carbon fixation processes, the RuBisCO (Foyer, Neukermans, Queval, Noctor, & Harbinson, 2012) shows no changes in the shallow plants. Moreover, contrarily to expectations, deep plants that would need to increase RuBisCO efficiency and carbon uptake under high light (Lambers, Chapin, & Pons, 2008) showed a trend toward its down‐regulation from T1 to T3. The sharp leaf‐respiratory increase displayed only by deep plants at the end of their exposure to high light responds to substrate supply and energy demand needed for leaf growth and maintenance (Lambers et al., 2008). It could also be affected by the increase in photorespiration (Atkin, Evans, Ball, Lambers, & Pons, 2000; Wingler et al., 2016) as also suggested by similarity in the expression profile of phosphoglycolate phosphatase between DS and SS (high light), while it was significantly different between SD and DD (low light). This enzyme is involved in the photorespiration cycle (Anderson, 1971; Bowes, Ogren, & Hageman, 1971; Ogren, 1984; Wingler et al., 2016), where it removes the toxic molecule 2‐phosphoglycolate (2‐PG) formed by the RuBisCO oxygenase activity. Besides, only slight variations of phosphoglycolate phosphatase levels were displayed among each couple of test and control groups (SD, SS and DS, DD), suggesting that photorespiration rate may be a fixed trait between stands.

The increased leaf sugar content of deep plants likely induced the increase in respiration, to sustain a faster growth as well as the increased demand for energy that occurs under high light (e.g., increased rates of biosynthesis and protein turnover). Shallow plants seem to be unable to acclimate respiration under low light (i.e., with reduced substrate supply), resulting in reduced carbohydrate content, with potential impact on plant growth and long‐term survival. The negative impact of the reduction of energetic compounds associated with light reduction was previously reported in a shading experiment in the field (Ruiz & Romero, 2001). Nevertheless, we did not record a significant mortality, probably related to the fact that plants were collected during late summer (i.e., the season of maximum sugar concentration), when their carbohydrates reserves might have supported growth and survival during the experimental shading period (Alcoverro, Cerbian, & Ballesteros, 2001).

Results of phenotypic clustering indicated that at the end of the exposition to reciprocal light conditions, plants of both tester groups were better regrouped on the basis of their origin (axis 1 of gene expression and photo‐physiology, >57% of variance) rather than the light environment at which they are exposed. This suggests that most of the traits analyzed are genetically fixed in the two stands and that phenotypes are plastic only for few of them. The response to contrasting light conditions is achieved through distinct photo‐adaptive strategies, and plants from different light environments do not converge when exposed to the same light treatment.

Several studies indicated that gene expression variation has an important role in evolutionary processes of adaptive divergence among natural populations (Derome, Duchesne, & Bernatchez, 2006; Filiault & Maloof, 2012; Granados‐Cifuentes et al., 2013; Larsen et al., 2007; Oleksiak, Churchill, & Crawford, 2002; Oleksiak, Roach, & Crawford, 2005; Sultan, 2000) due to the high heritability of gene regulation (Pavey et al., 2010; Roelofs, Mariën, & van Straalen, 2007; Schadt et al., 2003; Whitehead & Crawford, 2006).

Here photo‐physiological acclimation to light is supported by the plasticity of few target genes, while others show low plasticity in response to contrasting light. The differences observed in the number of light‐responsive genes as well as in their level of expression suggest that environmental selection operated on different traits to select the appropriate phenotype that best fits the light environment existing at the extremes of the bathymetrical distribution of the species. For example, shallow plants living under high light need to replace damaged components of the photosynthetic apparatus in order to maintain photosynthetic functioning (Muramatsu & Hihara, 2012; Walters, 2005). Accordingly, they showed a general up‐regulation of structural components of PSI and PSII and light‐harvesting proteins with respect to deep ones.

Genes involved in the processes of photosynthesis (i.e., LHCs), photoreception (in particular PHYA and CRY1), and carbon fixation (i.e., RbcS and PGLP) accounted for the higher contributions to the differentiation among the four experimental groups, and their expression profiles were more correlated with the plant's origin than with the new light environment.

Environmental light sensing, in particular, seems to be constitutively different between deep and shallow clones, and “Photoreceptors” and “Photoperiodic” genes seem to have a central role in the adaptive diversification of *P. oceanica* along the depth cline. Indeed, photoreceptors are involved in evolutionary processes influencing natural selection of phenotypic traits (Mitchell‐Olds, Willis, & Goldstein, 2007). Both phytochromes and cryptochromes mediate several morphological, physiological, and developmental responses to red, far‐red, and blue light in plants and algae (Li & Yang, 2007; Maloof, Borevitz, Weigel, & Chory, 2000; Schmitt, Stinchcombe, Heschel, & Huber, 2003; Smith, 2000). They have also a key role in controlling photoperiod in plants (Balasubramanian et al., 2006; Hall et al., 2007; Mendez‐Vigo, Pico, Ramiro, & Martı, 2011; Slotte, Holm, Mcintyre, Lagercrantz, & Lascoux, 2007). Indeed, the down‐regulation of cryptochrome 1 (during the whole exposure period) and phytochrome A (in T2 and T3) is noteworthy because these two photoreceptors are intimately involved in promoting several light acclimation responses in higher plants (see, e.g., Walters, Rogers, Shephard, & Horton, 1999).

The adaptive variation of photoreceptors and photoperiod genes could also explain the difference in flowering and reproductive times detected along the depth gradient in *P. oceanica* (Buia & Mazzella, 1991). This shift in the reproductive phenology reduces gene flow along the depth, enhancing the partial isolation and neutral drift of the different meadow's stands (D'Esposito et al., 2012; Migliaccio et al., 2005; Procaccini & Mazzella, 1998; Procaccini et al., 2001). In addition, despite the potential free dispersion of clonal fragments within the meadow, the strong separation between shallow and deep stands indicates that recruitment of adult individuals coming from other depths is rare. Multiple isolating mechanisms (Nosil et al., 2005) may operate along the depth gradient by not only promoting the reproductive isolation but also affecting the survival of immigrants. Although in our experiment clones collected at different depths showed phenotypic buffering and partial acclimation to the reciprocal light conditions in the experimental time frame, we must consider that light is only one of the environmental factors changing between different depths. Temperature has also been analyzed as another environmental factor driving adaptive divergence between shallow and deep portions of the same meadow (Marín‐Guirao et al., 2016), and we cannot exclude that on a long run phenotype–environment mismatches (Marshall et al., 2010) would prevent effective recruitment. Moreover, the process of “matching habitat choice” (Edelaar et al., 2008) might reduce the reproductive success of immigrant clones in relation to local ones. Collectively, our data suggest that ecological selection (Rundle & Nosil, 2005; Schluter, 2009) plays a role in the genetic diversification of *P. oceanica* populations along depth. Indeed, adaptive divergence at phenotypic level can facilitate the increase in neutral genetic diversity through isolation by adaptation (Nosil et al., 2008). More studies are needed to clarify the interactions among neutral divergence, interindividual interactions, and mating behavior along the depth cline (Doebeli & Dieckmann, 2000, 2003).

In conclusion, while the structure of genetic diversity among geographic regions of *P. oceanica* appears coherent with neutral processes (i.e., Serra et al., 2010), at local‐scale phenotypic plasticity, phenotypic buffering and adaptive divergence also play a role. We suggest that different portions of the same meadow, which are genetically distinct according to neutral markers, possess enough phenotypic buffering for partially acclimating to reciprocal light conditions, but retain the photo‐physiological imprinting of their original light environment. Shallow plants in low light conditions show a reduction in the energetic status that could be interpreted as an initial hint for maladaptation. Hence, we support the existence of adaptive divergence between the two meadow portions and we identified key processes and genes putatively involved in the process. We believe that the information provided by this study may enhance our understanding of the fine‐scale response of this key species to global climatic changes that involve changes in light and many other environmental drivers. Moreover, the information provided here may improve restoration strategies, stressing the importance to design criteria which take into account the suitability of the genetic pool of donor populations (Hufford & Mazer, 2003; Jahnke et al., 2015; van Katwijk et al., 2009; Procaccini & Piazzi, 2001; Vander Mijnsbrugge, Bischoff, & Smith, 2010) and more generally define the effect of phenotypic plasticity on populations viability (Wennersten & Forsman, 2012) beyond which there are potentially negative restoration consequences. Low plasticity in fundamental metabolic pathways and fixed phenotypic traits could impair the successful adaptation of foreign genetic material.

## Data accessibility

The reference transcriptome of *P. oceanica* is available at NCBI (Accession number: GEMD01000000). The raw matrix of microsatellite genotypes for all individuals is available at Suppl mat Table S7.

## Conflict of Interest

None declared.

## References

[ece32731-bib-0001] Agrawal, A. A. (2001). Phenotypic plasticity in the interactions and evolution of species. Science, 294(5541), 321–326. 11598291 10.1126/science.1060701

[ece32731-bib-0002] Alcoverro, T. , Cerbian, E. , & Ballesteros, E. (2001). The photosynthetic capacity of the seagrass *Posidonia oceanica*: Influence of nitrogen and light. Journal of Experimental Marine Biology and Ecology, 261, 107–120. 11438108 10.1016/s0022-0981(01)00267-2

[ece32731-bib-0003] Anderson, J. G. (1971). Rocket measurement of OH in the mesosphere. Journal of Geophysical Research, 76, 7820–7824.

[ece32731-bib-0004] Arnaud‐Haond, S. , Duarte, C. M. , Diaz‐Almela, E. , Marbà, N. , Sintes, T. , & Serrão, E. A. (2012). Implications of extreme life span in clonal organisms: millenary clones in meadows of the threatened seagrass *Posidonia oceanica* . PLoS One, 7(2), e30454. doi:10.1371/journal.pone.0030454. 22312426 10.1371/journal.pone.0030454 PMC3270012

[ece32731-bib-0006] Atkin, O. K. , Evans, J. R. , Ball, M. C. , Lambers, H. , & Pons, T. L. (2000). Leaf respiration of snow gum in the light and dark. Interactions between Temperature and Irradiance. Plant Physiology, 122, 915–923. 10712556 10.1104/pp.122.3.915 PMC58928

[ece32731-bib-0007] Balasubramanian, S. , Sureshkumar, S. , Agrawal, M. , et al. (2006). The phytochrome c photoreceptor gene mediates natural variation in flowering and growth responses of Arabidopsis thaliana. Nature Genetics, 38, 711–715. 16732287 10.1038/ng1818 PMC1592229

[ece32731-bib-0008] Barbier, E. B. , Hacker, S. D. , Kennedy, C. , et al. (2011). The value of estuarine and coastal ecosystem services. Ecological Monographs, 81, 169–193.

[ece32731-bib-0009] Baythavong, B. S. (2011). Linking the spatial scale of environmental variation and the evolution of phenotypic plasticity: Selection favors adaptive plasticity in fine‐grained environments. The American Naturalist, 178, 75–87. 10.1086/660281 21670579

[ece32731-bib-0010] Bongaerts, P. , Frade, P. R. , Ogier, J. J. , et al. (2013). Sharing the slope: Depth partitioning of agariciid corals and associated Symbiodinium across shallow and mesophotic habitats (2‐60 m) on a Caribbean reef Sharing the slope: Depth partitioning of agariciid corals and associated Symbiodinium across shal. BMC Evloutionary Biology, 13, 205–218. 10.1186/1471-2148-13-205 PMC3849765 24059868

[ece32731-bib-0011] Bowes, G. , Ogren, W. L. , & Hageman, R. H. (1971). Phosphoglycolate production catalyzed by ribulose diphosphate carboxylase. Biochemical and Biophysical Research Communications, 45, 716–722. 4331471 10.1016/0006-291x(71)90475-x

[ece32731-bib-0012] Bricker, E. , Waycott, M. , Calladine, A. , & Zieman, J. C. (2011). High connectivity across environmental gradients and implications for phenotypic plasticity in a marine plant. Marine Ecology‐Progress Series, 423, 57–67.

[ece32731-bib-0013] Buia, M. C. , & Mazzella, L. (1991). Reproductive phenology of the Mediterranean seagrasses *Posidonia oceanica* (L.) *Delile*,* Cymodocea nodosa* (Ucria) Aschers., and *Zostera noltii* Hornem. Aquatic Botany, 40, 343–362.

[ece32731-bib-0014] Callahan, H. S. , Maughan, H. , & Steiner, U. K. (2008). Phenotypic plasticity, costs of phenotypes, and costs of plasticity: Toward an integrative view. Annals of the New York Academy of Sciences, 1133, 44–66. 18559815 10.1196/annals.1438.008

[ece32731-bib-0015] Carr, M. R. (1996). PRIMER user manual: Plymouth routines in multivariate ecological research. Plymouth Marine Laboratory.

[ece32731-bib-0016] Chevin, L.‐M. , Gallet, R. , Gomulkiewicz, R. , Holt, R. D. , & Fellous, S. (2013). Phenotypic plasticity in evolutionary rescue experiments. Philosophical Transactions of the Royal Society B: Biological Sciences, 368, 20120089. doi:10.1098/rstb.2012.0089. 10.1098/rstb.2012.0089 PMC3538455 23209170

[ece32731-bib-0017] Chevin, L.‐M. , Lande, R. , & Mace, G. M. (2010). Adaptation, plasticity, and extinction in a changing environment: towards a predictive theory. PLoS Biology, 8(4), e1000357. doi:10.1371/journal.pbio.1000357. 20463950 10.1371/journal.pbio.1000357 PMC2864732

[ece32731-bib-0018] Clarke, K. R. , & Gorley, R. N. (2001). PRIMER v6. Plymouth, UK: PRIMER‐E Ltd.

[ece32731-bib-0019] Collier, C. J. , Lavery, P. S. , Ralph, P. J. , & Masini, R. J. (2008). Physiological characteristics of the seagrass *Posidonia sinuosa* along a depth‐related gradient of light availability. Marine Ecology‐Progress Series, 353, 65–79.

[ece32731-bib-0020] Conover, D. O. , Duffy, T. A. , & Hice, L. A. (2009). The covariance between genetic and environmental influences across ecological gradients: Reassessing the evolutionary significance of countergradient and cogradient variation. Annals of the New York Academy of Sciences, 1168, 100–129. 19566705 10.1111/j.1749-6632.2009.04575.x

[ece32731-bib-0021] Costanza, R. , D'Arge, R. , De Groot, R. , et al. (1997). The value of the world's ecosystem services and natural capital. Nature, 387, 253–260.

[ece32731-bib-0022] Cowen, R. K. , & Sponaugle, S. (2009). Larval dispersal and marine population connectivity. Annual Review of Marine Science, 1, 443–466. 10.1146/annurev.marine.010908.163757 21141044

[ece32731-bib-0023] Dalla Via, J. , Sturmbauer, C. , Schonweger, G. , et al. (1998). Light gradients and meadow structure in *Posidonia oceanica*: Ecomorphological and functional correlates. Marine Ecology Progress Series, 163, 267–278.

[ece32731-bib-0024] Dattolo, E. , Gu, J. , Bayer, P. E. , et al. (2013). Acclimation to different depths by the marine angiosperm *Posidonia oceanica*: Transcriptomic and proteomic profiles. Frontiers in Plant Science, 4, 195–199. 23785376 10.3389/fpls.2013.00195 PMC3683636

[ece32731-bib-0025] Dattolo, E. , Ruocco, M. , Brunet, C. , et al. (2014). Response of the seagrass *Posidonia oceanica* to different light environments: Insights from a combined molecular and photo‐physiological study. Marine Environmental Research, 101, 225–236. 25129449 10.1016/j.marenvres.2014.07.010

[ece32731-bib-0026] De Carvalho, L. , Chaves, T. , Ormond, C. G. A. , Mcginty, E. S. , & Ferreira, B. P. (2012). Space partitioning among damselfishes in the Caribbean coast of Panama: The role of habitat preferences. Neotropical Ichthyology, 10, 633–642.

[ece32731-bib-0027] Demmig‐Adams, B. , Adams, W. W. III , & Mattoo, A. K. (Eds.) (2006). Photoprotection, photoinhibition, gene regulation, and environment. Advances in photosynthesis and respiration, Vol 21. Dordrecht: Springer, Hardcover edition.

[ece32731-bib-0028] Derome, N. , Duchesne, P. , & Bernatchez, L. (2006). Parallelism in gene transcription among sympatric lake whitefish (*Coregonus clupeaformis Mitchill*) ecotypes. Moleculae Ecology, 15, 1239–1249. 10.1111/j.1365-294X.2005.02968.x 16626451

[ece32731-bib-0029] D'Esposito, D. , Dattolo, E. , Badalamenti, F. , Orsini, L. , & Procaccini, G. (2012). Comparative analysis of genetic diversity of *Posidonia oceanica* along a depth gradient using neutral and selective/non neutral microsatellites markers. Biologia Marina Mediterranea, 19, 45–48.

[ece32731-bib-0030] Doebeli, M. , & Dieckmann, U. (2000). Evolutionary branching and sympatric speciation caused by different types of ecological interactions. The American Naturalist, 156, S77–S101. 10.1086/303417 29592583

[ece32731-bib-0031] Doebeli, M. , & Dieckmann, U. (2003). Speciation along environmental gradients. Nature, 421, 259–264. 12529641 10.1038/nature01274

[ece32731-bib-0032] Dowdall, T. J. , Handelsman, C. A. , Ruell, E. W. , et al. (2012). Fine‐scale local adaptation in life histories along a continuous environmental gradient in Trinidadian guppies. Functional Ecology, 26, 616–627.

[ece32731-bib-0033] Duarte, C. M. (1991). Seagrass depth limits. Aquatic Botany, 40, 363–377.

[ece32731-bib-0034] Edelaar, P. , Siepielski, A. M. , & Clobert, J. (2008). Matching habitat choice causes directed gene flow: A neglected dimension in evolution and ecology. Evolution, 62, 2462–2472. 18637835 10.1111/j.1558-5646.2008.00459.x

[ece32731-bib-0035] Endler, J. A. (1977). Geographic variation, speciation, and clines. Princeton: Princeton University Press. 409931

[ece32731-bib-0036] Enríquez, S. (2005). Light absorption efficiency and the package effect in the leaves of the seagrass *Thalassia testudinum* . Marine Ecology Progress Series, 289, 141–150.

[ece32731-bib-0037] Evans, T. G. , & Hofmann, G. E. (2012). Defining the limits of physiological plasticity: How gene expression can assess and predict the consequences of ocean change. Philosophical Transactions of the Royal Society of London. Series B, Biological Sciences, 367, 1733–1745. 22566679 10.1098/rstb.2012.0019 PMC3350660

[ece32731-bib-0038] Filiault, D. L. , & Maloof, J. N. (2012). A genome‐wide association study identifies variants underlying the Arabidopsis thaliana shade avoidance response. PLoS Genetics, 8(3), e1002589. 22438834 10.1371/journal.pgen.1002589 PMC3305432

[ece32731-bib-0039] Forsman, A. (2014). Rethinking phenotypic plasticity and its consequences for individuals, populations and species. Heredity (Edinb), 115, 276–284. 25293873 10.1038/hdy.2014.92 PMC4815454

[ece32731-bib-0040] Foyer, C. H. , Neukermans, J. , Queval, G. , Noctor, G. , & Harbinson, J. (2012). Photosynthetic control of electron transport and the regulation of gene expression. Journal of Experimental Botany, 63, 1637–1661. 22371324 10.1093/jxb/ers013

[ece32731-bib-0041] Franks, S. J. , Sim, S. , & Weis, A. E. (2007). Rapid evolution of flowering time by an annual plant in response to a climate fluctuation. Proceedings of the National Academy of Sciences of the United States of America, 104, 1278–1282. 17220273 10.1073/pnas.0608379104 PMC1783115

[ece32731-bib-0042] Franks, S. J. , Weber, J. J. , & Aitken, S. N. (2014). Evolutionary and plastic responses to climate change in terrestrial plant populations. Evolutionary Applications, 7, 123–139. 24454552 10.1111/eva.12112 PMC3894902

[ece32731-bib-0043] García‐Ramos, G. , & Huang, Y. (2013). Competition and evolution along environmental gradients: Patterns, boundaries and sympatric divergence. Evolutionary Ecology, 27, 489–504.

[ece32731-bib-0044] Ghalambor, C. K. , McKay, J. K. , Carroll, S. P. , & Reznick, D. N. (2007). Adaptive versus non‐adaptive phenotypic plasticity and the potential for contemporary adaptation in new environments. Functional Ecology, 21, 394–407.

[ece32731-bib-0045] Gienapp, P. , Teplitsky, C. , Alho, J. S. , Mills, J. A. , & Merilä, J. (2008). Climate change and evolution: Disentangling environmental and genetic responses. Molecular Ecology, 17, 167–178. 18173499 10.1111/j.1365-294X.2007.03413.x

[ece32731-bib-0046] Granados‐Cifuentes, C. , Bellantuono, A. J. , Ridgway, T. , Hoegh‐Guldberg, O. , & Rodriguez‐Lanetty, M. (2013). High natural gene expression variation in the reef‐building coral *Acropora millepora*: Potential for acclimative and adaptive plasticity. BMC Genomics, 14, 228–242. 23565725 10.1186/1471-2164-14-228 PMC3630057

[ece32731-bib-0047] Hall, D. , Luquez, V. , Garcia, V. M. , et al. (2007). Adaptive population differentiation in phenology across a latitudinal gradient in European aspen (*Populus tremula, L*.): A comparison of neutral markers, candidate genes and phenotypic traits. Evolution, 61, 2849–2860. 17908247 10.1111/j.1558-5646.2007.00230.x

[ece32731-bib-0048] Hammer, Ø. , Harper, D. A. T. , & Ryan, P. D. (2001). PAST: Paleontological statistics software package for education and data analysis. Palaeontologia Electronica, 4, 1–9.

[ece32731-bib-0049] Hemminga, M. A. , & Duarte, C. M. (2000). Seagrass ecology. Cambridge: Cambridge University Press.

[ece32731-bib-0050] Hice, L. A. , Duffy, A. T. , Munch, B. S. , & Conover, O. D. (2012). Spatial scale and divergent patterns of variation in adapted traits in the ocean. Ecology Letters, 15, 568–575. 22462779 10.1111/j.1461-0248.2012.01769.x

[ece32731-bib-0051] Hoffmann, A. A. , & Sgro, C. M. (2011). Climate change and evolutionary adaptation. Nature, 470, 479–485. 21350480 10.1038/nature09670

[ece32731-bib-0052] Hufford, K. M. , & Mazer, S. J. (2003). Plant ecotypes: Genetic differentiation in the age of ecological restoration. Trends in Ecology & Evolution, 18, 147–155.

[ece32731-bib-0053] Hughes, A. R. , Williams, S. L. , Duarte, C. M. , Heck, K. L. , & Waycott, M. (2009). Associations of concern: Declining seagrasses and threatened dependent species. Frontiers in Ecology and the Environment, 7, 242–246.

[ece32731-bib-0054] Jahnke, M. , Olsen, J. L. , & Procaccini, G. (2015). A meta‐analysis reveals a positive correlation between genetic diversity metrics and environmental status in the long‐lived seagrass *Posidonia oceanica* . Molecular Ecology, 24, 2336–2348. 25819368 10.1111/mec.13174

[ece32731-bib-0055] Jeukens, J. , Bittner, D. , Knudsen, R. , & Bernatchez, L. (2009). Candidate genes and adaptive radiation: Insights from transcriptional adaptation to the limnetic niche among coregonine fishes (*Coregonus* spp., Salmonidae). Molecular Biology and Evolution, 26, 155–166. 18927090 10.1093/molbev/msn235

[ece32731-bib-0056] van Katwijk, M. M. , Bos, A. R. , de Jonge, V. N. , et al. (2009). Guidelines for seagrass restoration: Importance of habitat selection and donor population, spreading of risks, and ecosystem engineering effects. Marine Pollution Bulletin, 58, 179–188. 19131078 10.1016/j.marpolbul.2008.09.028

[ece32731-bib-0057] Kawecki, T. J. , & Ebert, D. (2004). Conceptual issues in local adaptation. Ecology Letters, 7, 1225–1241.

[ece32731-bib-0058] Keller, I. , & Seehausen, O. (2012). Thermal adaptation and ecological speciation. Molecular Ecology, 21, 782–799. 22182048 10.1111/j.1365-294X.2011.05397.x

[ece32731-bib-0059] Kirk, J. T. O. (2011). Light and photosynthesis in aquatic ecosystems. New York: Cambridge university press.

[ece32731-bib-0060] Lambers, H. , Chapin, F. S. III , & Pons, T. L. (2008). Photosynthesis In Plant physiological ecology (pp. 11–99). New York: Springer.

[ece32731-bib-0061] Larkum, A. W. D. , & Orth, R. J. (2014). Book review – Seagrasses: Biology, ecology and conservation. IMarine Ecology, 20, 266–267.

[ece32731-bib-0062] Larsen, P. F. , Nielsen, E. E. , Williams, T. D. , & Loeschcke, V. (2008). Intraspecific variation in expression of candidate genes for osmoregulation, heme biosynthesis and stress resistance suggests local adaptation in European flounder (*Platichthys flesus*). Heredity, 101, 247–259. 18560442 10.1038/hdy.2008.54

[ece32731-bib-0063] Larsen, P. F. , Nielsen, E. E. , Williams, T. D. , et al. (2007). Adaptive differences in gene expression in European flounder (*Platichthys flesus*). Molecular Ecology, 16, 4674–4683. 17922814 10.1111/j.1365-294X.2007.03530.x

[ece32731-bib-0064] Lauritano, C. , Ruocco, M. , Dattolo, E. , et al. (2015). Response of key stress‐related genes of the seagrass *Posidonia oceanica* in the vicinity of submarine volcanic vents. Biogeosciences, 12, 4185–4194.

[ece32731-bib-0065] Li, Q.‐H. , & Yang, H.‐Q. (2007). Cryptochrome signaling in plants. Photochemistry and Photobiology, 83, 94–101. 17002522 10.1562/2006-02-28-IR-826

[ece32731-bib-0066] Lichtenthaler, H. K. , & Wellburn, A. R. (1983). Determinations of total carotenoids and chlorophylls a and b of leaf extracts in different solvents. Biochemical Society Transactions, 11, 591–592.

[ece32731-bib-0067] Maloof, J. N. , Borevitz, J. O. , Weigel, D. , & Chory, J. (2000). Natural variation in phytochrome signaling. Seminars in Cell & Developmental Biology, 11, 523–530. 11145882 10.1006/scdb.2000.0198

[ece32731-bib-0068] Marín‐Guirao, L. , Ruiz, J. M. , Dattolo, E. , Garcia‐Munoz, R. , & Procaccini, G. (2016). Physiological and molecular evidence of differential short‐term heat tolerance in Mediterranean seagrasses. Scientific Reports, 6, 28615. 27345831 10.1038/srep28615 PMC4921816

[ece32731-bib-0069] Marín‐Guirao, L. , Ruiz, J. M. , & Sandoval‐Gil, J. M. (2015). Resistance of *Posidonia oceanica* seagrass meadows to the spread of the introduced green alga *Caulerpa cylindracea*: Assessment of the role of light. Biological Invasions, 17, 1989–2009.

[ece32731-bib-0070] Marín‐Guirao, L. , Ruiz, J. M. , Sandoval‐Gil, J. M. , et al. (2013). Xanthophyll cycle‐related photoprotective mechanism in the Mediterranean seagrasses *Posidonia oceanica* and *Cymodocea nodosa* under normal and stressful hypersaline conditions. Aquatic Botany, 109, 14–24.

[ece32731-bib-0071] Marín‐Guirao, L. , Sandoval‐Gil, J. M. , Ruíz, J. M. , & Sánchez‐Lizaso, J. L. (2011). Photosynthesis, growth and survival of the Mediterranean seagrass *Posidonia oceanica* in response to simulated salinity increases in a laboratory mesocosm system. Estuarine, Coastal and Shelf Science, 92, 286–296.

[ece32731-bib-1111] Marín‐Guirao, L. , Sandoval‐Gil, J. M. , Bernardeau‐Esteller, J. , Ruíz, J. M. , & Sánchez‐Lizaso, J. L. (2013). Responses of the Mediterranean seagrass *Posidonia oceanica* to hypersaline stress duration and recovery. Marine Environmental Research, 84, 60–75. 23306019 10.1016/j.marenvres.2012.12.001

[ece32731-bib-0072] Marshall, D. , Monro, K. , Bode, M. , Keough, M. , & Swearer, S. (2010). Phenotype – environment mismatches reduce connectivity in the sea. Ecology Letters, 13, 128–140. 19968695 10.1111/j.1461-0248.2009.01408.x

[ece32731-bib-0073] Mazzuca, S. , Björk, M. , Beer, S. , et al. (2013). Establishing research strategies, methodologies and technologies to link genomics and proteomics to seagrass productivity, community metabolism, and ecosystem carbon fluxes. Frontiers in Plant Science, 4, 1–19. 23515425 10.3389/fpls.2013.00038 PMC3601598

[ece32731-bib-0074] McMahon, K. , Collier, C. , & Lavery, P. S. (2013). Identifying robust bioindicators of light stress in seagrasses: A meta‐analysis. Ecological Indicators, 30, 7–15.

[ece32731-bib-0075] Mendez‐Vigo, B. , Pico, F. X. , Ramiro, M. , & Martı, M. (2011). Altitudinal and climatic adaptation is mediated by flowering traits and FRI, FLC, and PHYC genes in Arabidopsis. Plant Physiology, 157, 1942–1955. 21988878 10.1104/pp.111.183426 PMC3327218

[ece32731-bib-0076] Merilä, J. , & Hendry, A. P. (2014). Climate change, adaptation, and phenotypic plasticity: The problem and the evidence. Evolutionary Applications, 7, 1–14. 24454544 10.1111/eva.12137 PMC3894893

[ece32731-bib-0077] Migliaccio, M. , De Martino, F. , Silvestre, F. , & Procaccini, G. (2005). Meadow‐scale genetic structure in *Posidonia oceanica* . Marine Ecology‐Progress Series, 304, 55–65.

[ece32731-bib-0078] Mitchell‐Olds, T. , Willis, J. H. , & Goldstein, D. B. (2007). Which evolutionary processes influence natural genetic variation for phenotypic traits? Nature Reviews Genetics, 8, 845–856. 10.1038/nrg2207 17943192

[ece32731-bib-0005] Molecular Ecology Resources Primer Development Consortium , Arranz, S. E. , Avarre, J.‐C. , Balasundaram, C. , Bouza, C. , Calcaterra, N. B. , … Zhuang, Z. (2013). Permanent Genetic Resources added to Molecular Ecology Resources Database 1 December 2012–31 January 2013. Molecular Ecology Resources, 13, 546–549. doi:10.1111/1755‐0998.12095. 23521844 10.1111/1755-0998.12095

[ece32731-bib-0079] Muramatsu, M. , & Hihara, Y. (2012). Acclimation to high‐light conditions in cyanobacteria: From gene expression to physiological responses. Journal of Plant Research, 125, 11–39. 22006212 10.1007/s10265-011-0454-6

[ece32731-bib-0080] Nicotra, A. B. , Atkin, O. K. , Bonser, S. P. , et al. (2010). Plant phenotypic plasticity in a changing climate. Trends in Plant Science, 15, 684–692. 20970368 10.1016/j.tplants.2010.09.008

[ece32731-bib-0081] Niyogi, K. K. (1999). Photoprotection revisited: Genetic and molecular approaches. Annual Review of Plant Physiology Plant Nolecular Biology, 50, 333–359. 10.1146/annurev.arplant.50.1.333 15012213

[ece32731-bib-0082] Niyogi, K. K. (2000). Safety valves for photosynthesis. Current Opinion in Microbiology, 3, 455–460. 10.1016/s1369-5266(00)00113-8 11074375

[ece32731-bib-0083] Niyogi, K. K. , Li, X.‐P. , Rosenberg, V. , & Jung, H.‐S. (2005). Is PsbS the site of non‐photochemical quenching in photosynthesis? Journal of Experimental Botany, 56, 375–382. 15611143 10.1093/jxb/eri056

[ece32731-bib-1002] Nosil, P. , Vines, T. H. , & Funk, D. J. (2005). Perspective: reproductive isolation caused by natural selection against immigrants from divergent habitats. Evolution, 59, 705–719. 15926683

[ece32731-bib-0084] Nosil, P. , Egan, S. P. , & Funk, D. J. (2008). Heterogeneous genomic differentiation between walking‐stick ecotypes: “isolation by adaptation” and multiple roles for divergent selection. Evolution, 62, 316–336. 17999721 10.1111/j.1558-5646.2007.00299.x

[ece32731-bib-0085] Ogren, W. L. (1984). Photorespiration: Pathways, regulation, and modification. Annual Review of Plant Physiology, 35, 415–442.

[ece32731-bib-0086] Okonechnikov, K. , Golosova, O. , Fursov, M. , et al. (2012). Unipro UGENE: A unified bioinformatics toolkit. Bioinformatics, 28, 1166–1167. 22368248 10.1093/bioinformatics/bts091

[ece32731-bib-0087] Oleksiak, M. F. , Churchill, G. A. , & Crawford, D. L. (2002). Variation in gene expression within and among natural populations. Nature Genetics, 32, 261–266. 12219088 10.1038/ng983

[ece32731-bib-0088] Oleksiak, M. F. , Roach, J. L. , & Crawford, D. L. (2005). Natural variation in cardiac metabolism and gene expression in Fundulus heteroclitus. Nature Genetics, 37, 67–72. 15568023 10.1038/ng1483 PMC1447534

[ece32731-bib-0089] Olesen, B. , Enriquez, S. , Duarte, C. M. , & Sand‐Jensen, K. (2002). Depth‐acclimation of photosynthesis, morphology and demography of *Posidonia oceanica* and *Cymodocea nodosa* in the Spanish Mediterranean Sea. Marine Ecology‐Progress Series, 236, 89–97.

[ece32731-bib-0090] Orr, H. A. , & Unckless, R. L. (2008). Population extinction and the genetics of adaptation. The American Naturalist, 172, 160–169. 10.1086/589460 18662122

[ece32731-bib-0091] Orth, R. J. , Carruthers, T. J. B. , Dennison, W. C. , et al. (2006). A Global Crisis for Seagrass Ecosystems. BioScience, 56, 987–996.

[ece32731-bib-0092] Parmesan, C. , & Yohe, G. (2003). A globally coherent fingerprint of climate change impacts across natural systems. Nature, 421, 37–42. 12511946 10.1038/nature01286

[ece32731-bib-0093] Pavey, S. A. , Collin, H. , Nosil, P. , & Rogers, S. M. (2010). The role of gene expression in ecological speciation. Annals of the New York Academy of Sciences, 1206, 110–129. 20860685 10.1111/j.1749-6632.2010.05765.x PMC3066407

[ece32731-bib-0094] Peakall, R. , & Smouse, P. (2012). GenAlEx 6.5: Genetic analysis in Excel. Population genetic software for teaching and research‐an update. Bioinformatics, 28, 2537–2539. 22820204 10.1093/bioinformatics/bts460 PMC3463245

[ece32731-bib-0095] Perry, A. L. , Low, P. J. , Ellis, J. R. , & Reynolds, J. D. (2005). Climatic change and distribution shifts in marine fishes. Science, 308, 1912–1915. 15890845 10.1126/science.1111322

[ece32731-bib-0096] Pfaffl, M. W. , Horgan, G. W. , & Dempfle, L. (2002). Relative expression software tool (REST©) for group‐wise comparison and statistical analysis of relative expression results in real‐time PCR. Nucleic Acids Research, 30(9), e36. 11972351 10.1093/nar/30.9.e36 PMC113859

[ece32731-bib-0097] Pfennig, D. W. , Wund, M. A. , Snell‐Rood, E. C. , et al. (2010). Phenotypic plasticity's impacts on diversification and speciation. Trends in Ecology & Evolution, 25, 459–467. 20557976 10.1016/j.tree.2010.05.006

[ece32731-bib-0098] Pigliucci, M. (2001). Phenotypic plasticity: Beyond nature and nurture. Baltimore: JHU Press.

[ece32731-bib-0099] Pirc, H. (1986). Seasonal aspects of photosynthesis in *Posidonia oceanica*: Influence of depth, temperature and light intensity. Aquatic Botany, 26, 203–212.

[ece32731-bib-0100] Procaccini, G. , & Mazzella, L. (1998). Population genetic structure and gene flow in the seagrass *Posidonia oceanica* assessed using microsatellite analysis. Marine Ecology‐Progress Series, 169, 133–141.

[ece32731-bib-0101] Procaccini, G. , Orsini, L. , Ruggiero, M. V. , & Scardi, M. (2001). Spatial patterns of genetic diversity in *Posidonia oceanica* an endemic Mediterranean seagrass. Molecular Ecology, 10, 1413–1421. 11412364 10.1046/j.1365-294x.2001.01290.x

[ece32731-bib-0102] Procaccini, G. , & Piazzi, L. (2001). Genetic polymorphism and transplantation success in the mediterranean seagrass *Posidonia oceanica* . Restoration Ecology, 9, 332–338.

[ece32731-bib-0103] Quinn, G. P. , & Keough, M. J. (2002). Experimental design and data analysis for biologists. Cambridge: Cambridge University Press.

[ece32731-bib-0104] Ralph, P. J. , Durako, M. J. , Enriquez, S. , Collier, C. J. , & Doblin, M. A. (2007). Impact of light limitation on seagrasses. Journal of Experimental Marine Biology and Ecology, 350, 176–193.

[ece32731-bib-0105] Reusch, T. B. H. (2013). Climate change in the oceans: Evolutionary versus phenotypically plastic responses of marine animals and plants. Evolutionary Applications, 7, 104–122. 24454551 10.1111/eva.12109 PMC3894901

[ece32731-bib-0106] Reusch, T. B. H. , Boström, C. , Stam, W. T. , & Olsen, J. L. (1999). An ancient eelgrass clone in the Baltic. Marine Ecology Progress Series, 183, 301–304.

[ece32731-bib-0107] Reusch, T. B. H. , & Wood, T. E. (2007). Molecular ecology of global change. Molecular Ecology, 16, 3973–3992. 17894755 10.1111/j.1365-294X.2007.03454.x

[ece32731-bib-0108] Richardson, J. L. , Urban, M. C. , Bolnick, D. I. , & Skelly, D. K. (2014). Microgeographic adaptation and the spatial scale of evolution. Trends in Ecology & Evolution, 29, 165–176. 24560373 10.1016/j.tree.2014.01.002

[ece32731-bib-0109] Robakowski, P. , Li, Y. , & Reich, P. B. (2012). Local ecotypic and species range‐related adaptation influence photosynthetic temperature optima in deciduous broadleaved trees. Plant Ecology, 213, 113–125.

[ece32731-bib-0110] Roelofs, D. , Mariën, J. , & van Straalen, N. M. (2007). Differential gene expression profiles associated with heavy metal tolerance in the soil insect *Orchesella cincta* . Insect Biochemistry and Molecular Biology, 37, 287–295. 17368192 10.1016/j.ibmb.2006.11.013

[ece32731-bib-0111] Rozen, S. , & Skaletsky, H. (2000). Primer3 on the WWW for general users and for biologist programmers. Methods in Molecular Biology, 132, 365–386. 10547847 10.1385/1-59259-192-2:365

[ece32731-bib-0112] Rozenfeld, A. F. , Arnaud‐Haond, S. , Hernández‐García, E. , et al. (2007). Spectrum of genetic diversity and networks of clonal organisms. Journal of the Royal Society, Interface/the Royal Society, 4, 1093–1102. 10.1098/rsif.2007.0230 PMC2396204 17472906

[ece32731-bib-0113] Ruiz, J. M. , & Romero, J. (2001). Effects of in situ experimental shading on the Mediterranean seagrass *Posidonia oceanica* . Marine Ecology‐Progress Series, 215, 107–120.

[ece32731-bib-0114] Rundle, H. D. , & Nosil, P. (2005). Ecological speciation. Ecology Letters, 8, 336–352.

[ece32731-bib-0115] Sanford, E. , & Kelly, M. W. (2011). Local adaptation in marine invertebrates. Annual Review of Marine Science, 3, 509–535. 10.1146/annurev-marine-120709-142756 21329215

[ece32731-bib-0116] Savolainen, O. , Lascoux, M. , & Merilä, J. (2013). Ecological genomics of local adaptation. Nature reviews Genetics, 14, 807–820. 10.1038/nrg3522 24136507

[ece32731-bib-0117] Schadt, E. E. , Monks, S. A. , Drake, T. A. , et al. (2003). Genetics of gene expression surveyed in maize, mouse and man. Nature, 205, 1–6. 10.1038/nature01434 12646919

[ece32731-bib-0118] Scheiner, S. M. , & Lyman, F. (1989). The genetics of phenotypic plasticity. Journal of Evolutionary Biology, 107, 95–107.

[ece32731-bib-0119] Schlichting, C. D. (1986). The evolution of phenotypic plasticity in plants. Annual Review of Ecology and Systematics, 17, 667–693.

[ece32731-bib-0120] Schluter, D. (2009). Evidence for ecological speciation and its alternative. Science, 323, 737–741. 19197053 10.1126/science.1160006

[ece32731-bib-0121] Schmitt, J. , Stinchcombe, J. R. , Heschel, M. S. , & Huber, H. (2003). The adaptive evolution of plasticity: Phytochrome‐mediated shade avoidance responses. Integrative and Comparative Biology, 43, 459–469. 21680454 10.1093/icb/43.3.459

[ece32731-bib-0122] Sculthorpe, C. D. (1967). Biology of aquatic vascular plants. London: Edward Arnold.

[ece32731-bib-0123] Serra, I. A. , Innocenti, A. M. , Di Maida, G. , et al. (2010). Genetic structure in the Mediterranean seagrass *Posidonia oceanica*: Disentangling past vicariance events from contemporary patterns of gene flow. Molecular Ecology, 19, 557–568. 20051010 10.1111/j.1365-294X.2009.04462.x

[ece32731-bib-0124] Serra, I. A. , Lauritano, C. , Dattolo, E. , et al. (2012). Reference genes assessment for the seagrass *Posidonia oceanica* in different salinity, pH and light conditions. Marine Biology, 159, 1269–1282.

[ece32731-bib-0125] Short, F. T. , Polidoro, B. , Livingstone, S. R. , et al. (2011). Extinction risk assessment of the world's seagrass species. Biological Conservation, 144, 1961–1971.

[ece32731-bib-0126] Slotte, T. , Holm, K. , Mcintyre, L. M. , Lagercrantz, U. , & Lascoux, M. (2007). Differential Expression of Genes Important for Adaptation in Capsella bursa‐pastoris (Brassicaceae) 1 [W][OA]. Plant Physiology, 145, 160–173. 17631524 10.1104/pp.107.102632 PMC1976575

[ece32731-bib-0127] Smith, H. (2000). Phytochromes and light signal perception by plants‐an emerging synthesis. Nature, 407, 585–591. 11034200 10.1038/35036500

[ece32731-bib-0128] Sultan, S. E. (2000). Phenotypic plasticity for plant development, function and life history. Trends in Plant Science, 5, 537–542. 11120476 10.1016/s1360-1385(00)01797-0

[ece32731-bib-0129] Sultan, S. E. , & Bazzaz, F. A. (1993). Phenotypic plasticity in Polygonum persicaria. II. Norms of reaction to soil moisture and the maintenance of genetic diversity. Evolution, 47, 1032–1049. 10.1111/j.1558-5646.1993.tb02133.x 28564276

[ece32731-bib-0130] Swindell, W. R. , Huebner, M. , & Weber, A. P. (2007). Plastic and adaptive gene expression patterns associated with temperature stress in Arabidopsis thaliana. Heredity, 99, 143–150. 17473866 10.1038/sj.hdy.6800975

[ece32731-bib-0131] Telesca, L. , Belluscio, A. , Criscoli, A. , Ardizzone, G. , Apostolaki, E. T. , Fraschetti, S. , … Salomidi, M. (2015). Seagrass meadows (*Posidonia oceanica*) distribution and trajectories of change. Scientific Reports, 5. 10.1038/srep12505 PMC4516961 26216526

[ece32731-bib-0132] Thibert‐Plante, X. , & Hendry, A. P. (2011). The consequences of phenotypic plasticity for ecological speciation. Journal of Evolutionary Biology, 24, 326–342. 21091567 10.1111/j.1420-9101.2010.02169.x

[ece32731-bib-0133] Tomasello, A. , Di Maida, G. , Calvo, S. , Pirrotta, M. , Borra, M. , & Procaccini, G. (2009). Seagrass meadows at the extreme of environmental tolerance: the case of *Posidonia oceanica* in a semi‐enclosed coastal lagoon. Marine Ecology, 30(3), 288–300.

[ece32731-bib-0134] Underwood, A. J. (1997). Experiments in ecology: Their logical design and interpretation using analysis of variance. Cambridge: Cambridge University Press.

[ece32731-bib-0135] Valière, N. (2002). A computer program for analysing genetic GIMLET. Molecular Ecology Notes, 2, 377–379.

[ece32731-bib-0136] Valladares, F. , Matesanz, S. , Guilhaumon, F. , et al. (2014). The effects of phenotypic plasticity and local adaptation on forecasts of species range shifts under climate change. Ecology Letters, 17, 1351–1364. 25205436 10.1111/ele.12348

[ece32731-bib-0137] Van Kleunen, M. , & Fischer, M. (2005). Constraints on the evolution of adaptive phenotypic plasticity in plants. The New Phytologist, 166, 49–60. 15760350 10.1111/j.1469-8137.2004.01296.x

[ece32731-bib-0138] Vander Mijnsbrugge, K. , Bischoff, A. , & Smith, B. (2010). A question of origin: Where and how to collect seed for ecological restoration. Basic and Applied Ecology, 11, 300–311.

[ece32731-bib-0139] Walters, R. G. (2005). Towards an understanding of photosynthetic acclimation. Journal of Experimental Botany, 56, 435–447. 15642715 10.1093/jxb/eri060

[ece32731-bib-0140] Walters, R. G. , Rogers, J. J. M. , Shephard, F. , & Horton, P. (1999). Acclimation of *Arabidopsis thaliana* to the light environment: The role of photoreceptors. Planta, 209, 517–527. 10550634 10.1007/s004250050756

[ece32731-bib-0141] Waycott, M. , Duarte, C. M. , Carruthers, T. J. B. , et al. (2009). Accelerating loss of seagrasses across the globe threatens coastal ecosystems. Proceedings of the National Academy of Sciences of the United States of America, 106, 12377–12381. 19587236 10.1073/pnas.0905620106 PMC2707273

[ece32731-bib-0142] Wennersten, L. , & Forsman, A. (2012). Population‐level consequences of polymorphism, plasticity and randomized phenotype switching: A review of predictions. Biological Reviews, 87, 756–767. 22540928 10.1111/j.1469-185X.2012.00231.x

[ece32731-bib-0143] West‐Eberhard, M. J. (2005). Developmental plasticity and the origin of species differences. Proceedings of the National Academy of Sciences, 102, 6543–6549. 10.1073/pnas.0501844102 PMC1131862 15851679

[ece32731-bib-1003] Wissler, L. , Dattolo, E. , Moore, A. D. , Reusch, T. B. H. , Olsen, J. L. , Migliaccio, M. , & Procaccini, G. (2009). Dr. Zompo: An online data repository for *Zostera marina* and *Posidonia oceanica* ESTs. Database, 2009, bap009. 20157482 10.1093/database/bap009 PMC2790305

[ece32731-bib-0144] Whitehead, A. , & Crawford, D. L. (2006). Neutral and adaptive variation in gene expression. Proceedings of the National Academy of Sciences, 103, 5425–5430. 10.1073/pnas.0507648103 PMC1414633 16567645

[ece32731-bib-0145] Wingler, A. , Lea, P. J. , Quick, W. P. , et al. (2016). Photorespiration: Metabolic pathways and their role in stress protection. Philosophical Transactions of the Royal Society, 355, 1517–1529. 10.1098/rstb.2000.0712 PMC1692872 11128005

[ece32731-bib-0146] Zupo, V. , Mazzella, L. , Buia, M. C. , et al. (2006). A small‐scale analysis of the spatial structure of a *Posidonia oceanica* meadow off the Island of Ischia (Gulf of Naples, Italy): Relationship with the seafloor morphology. Aquatic Botany, 84, 101–109.

