# Supplementary figures and images for: Long‐term acclimation to reciprocal light conditions suggests depth‐related selection in the marine foundation species Posidonia oceanica
Source: Ecol Evol. 2017 Jan 24;7(4):1148–64. doi: 10.1002/ece3.2731 (PMC5306012; doi:10.1002/ece3.2731)

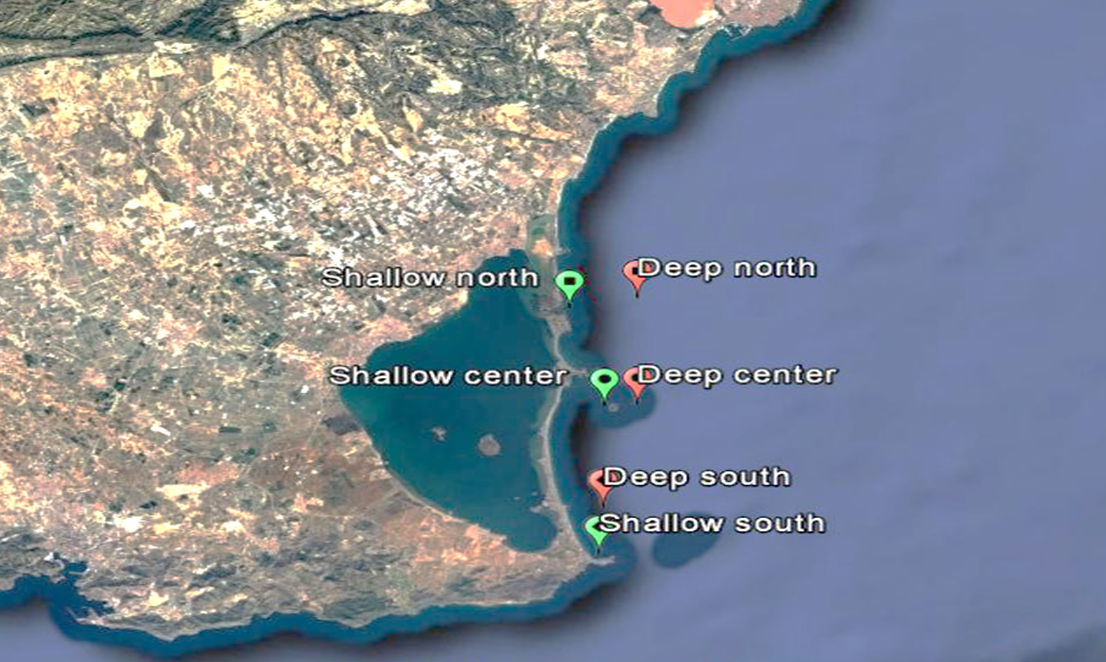

Supplement: Supplementary file 1 [file ECE3-7-1148-s001.tif]

**A**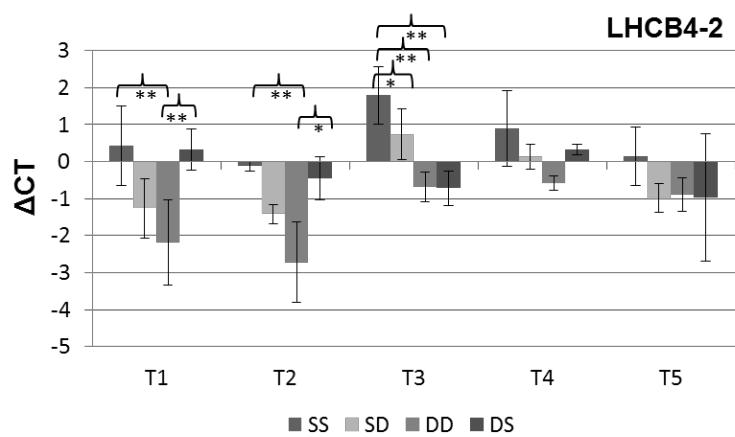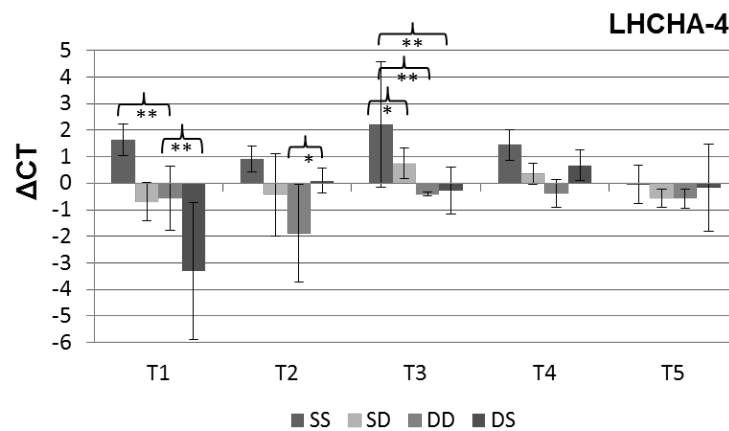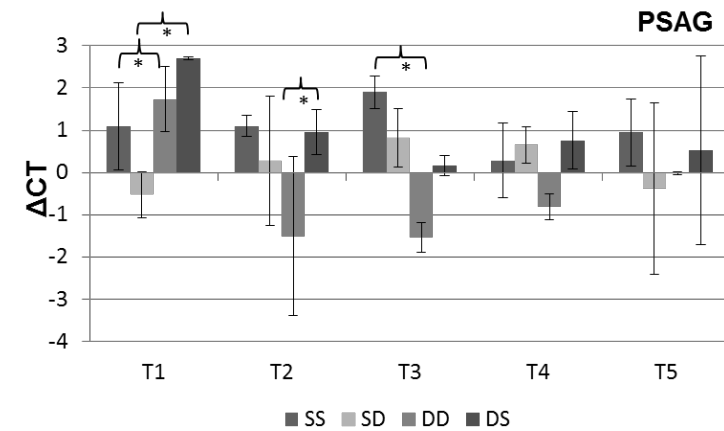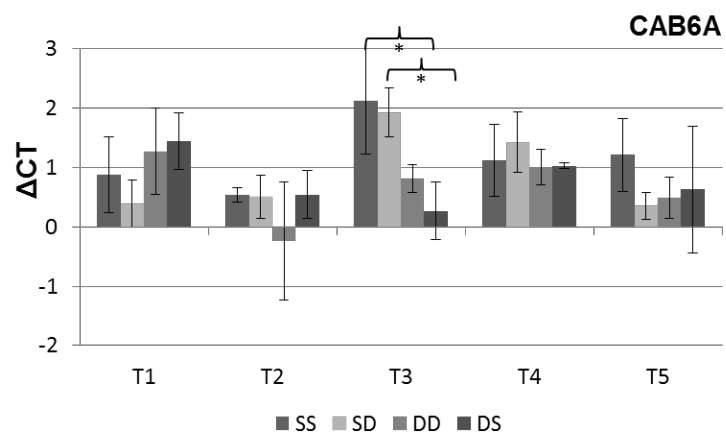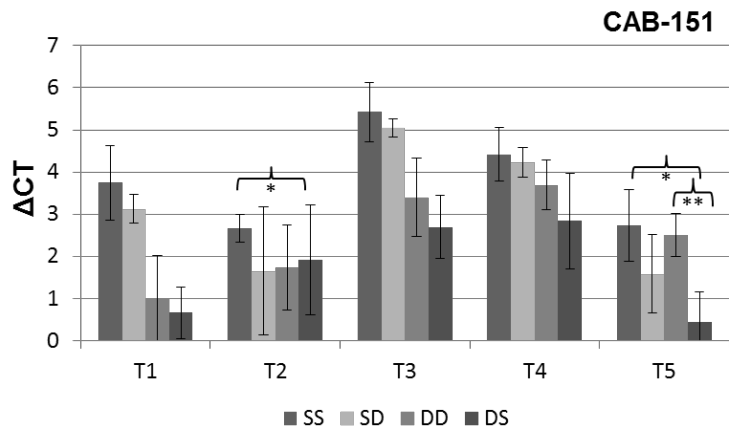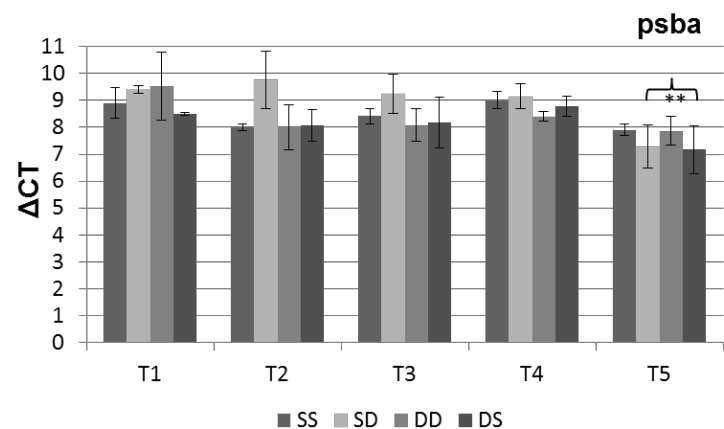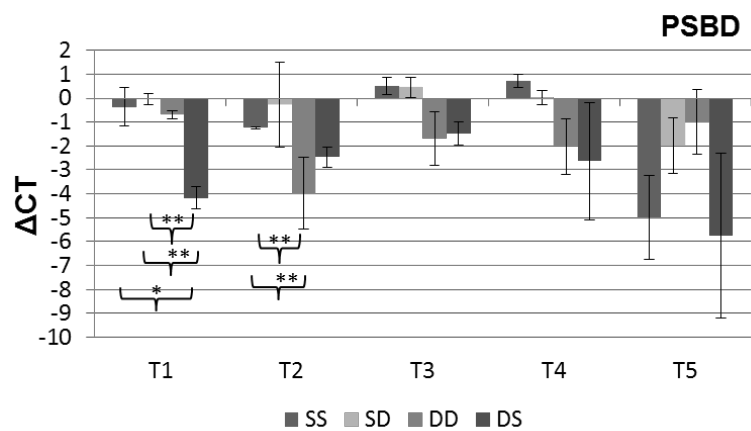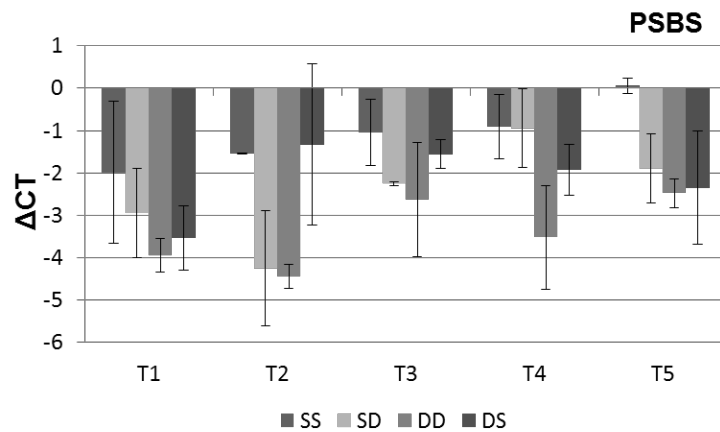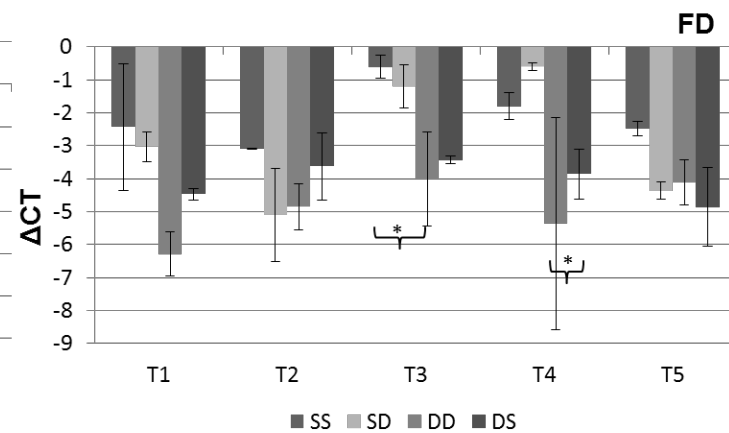

Supplement: Supplementary file 2 [file ECE3-7-1148-s002.pdf]

**B**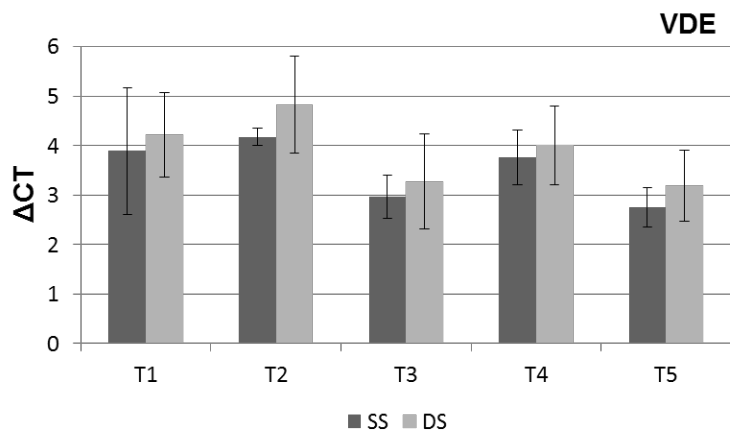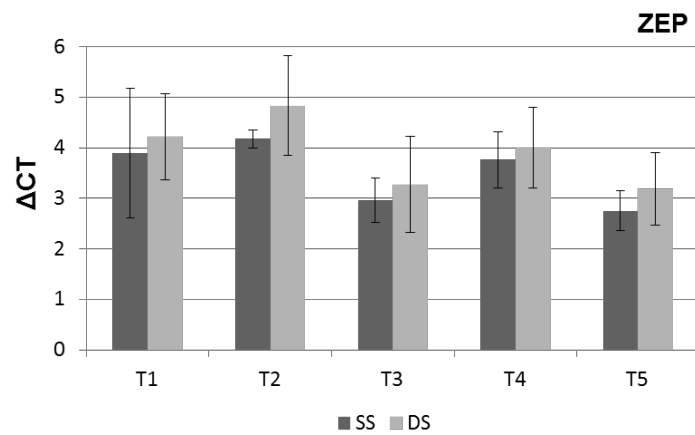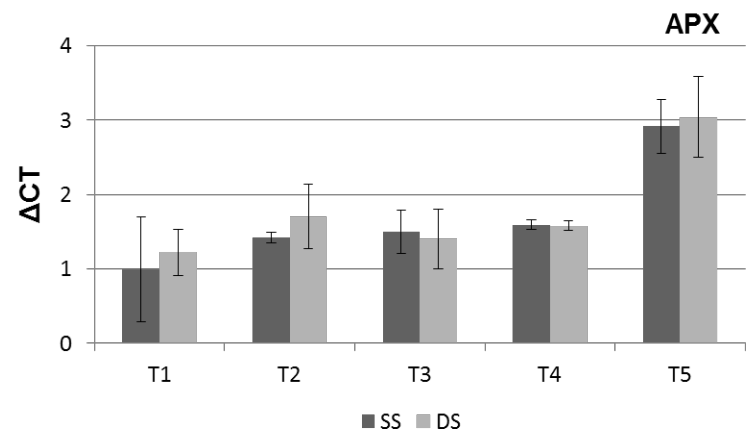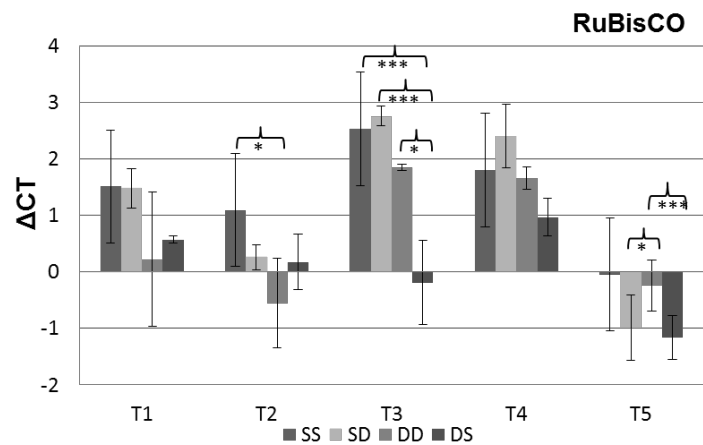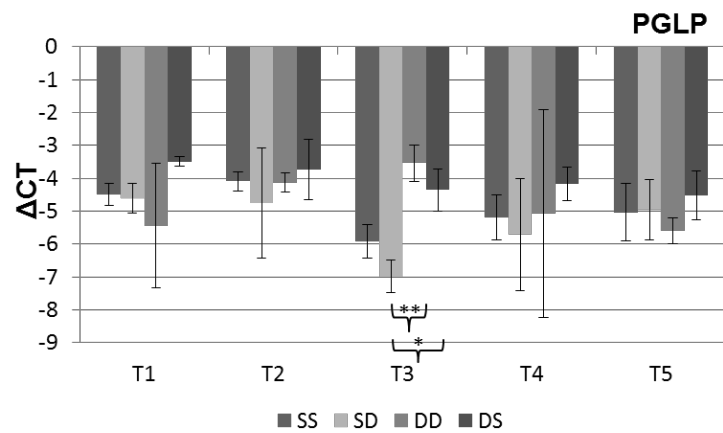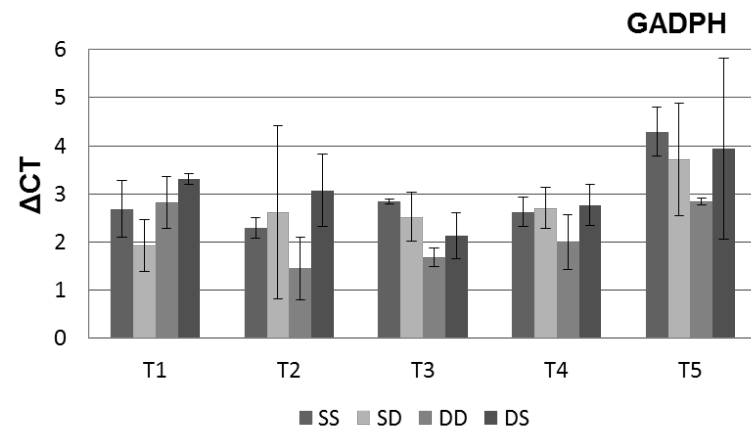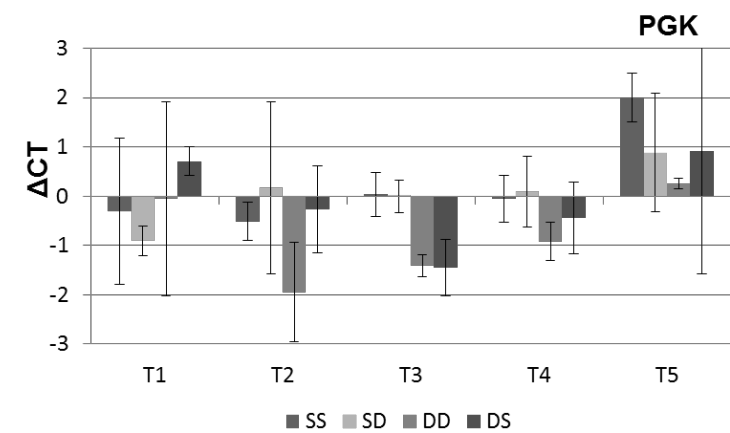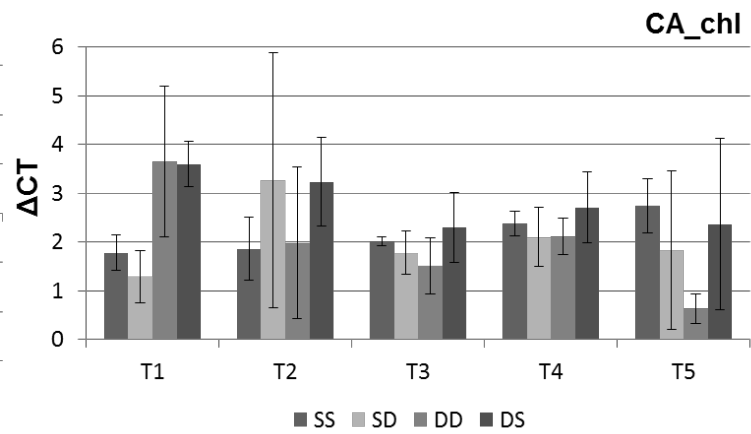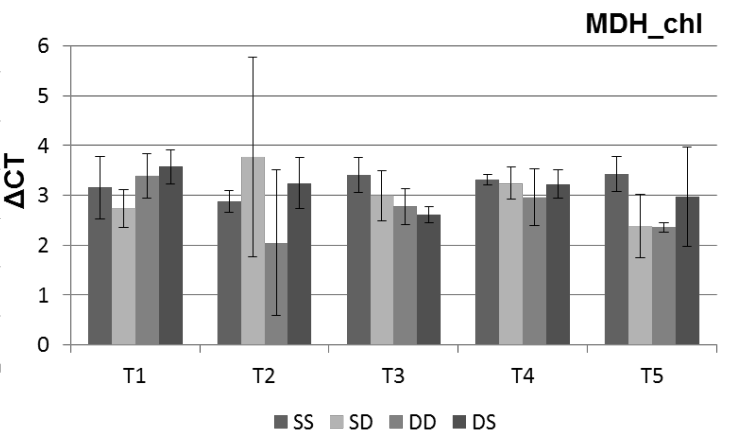

Supplement: Supplementary file 3 [file ECE3-7-1148-s003.pdf]

**C**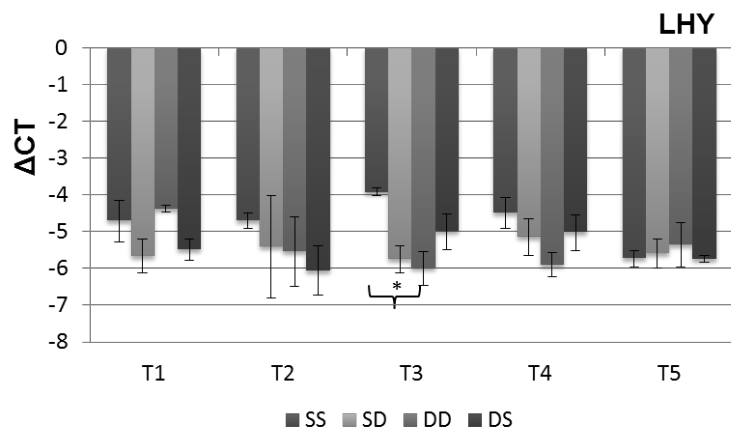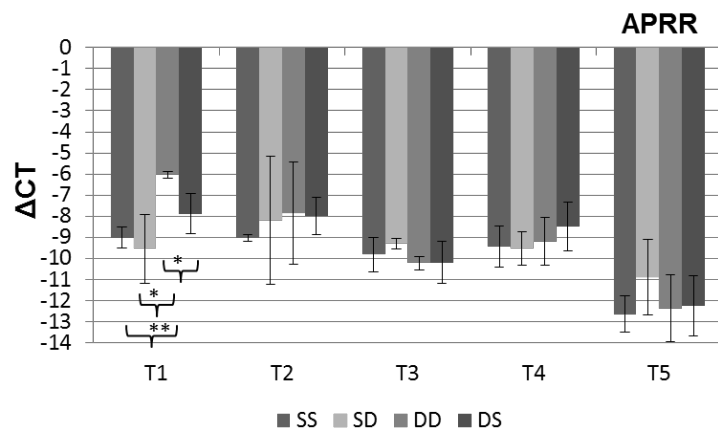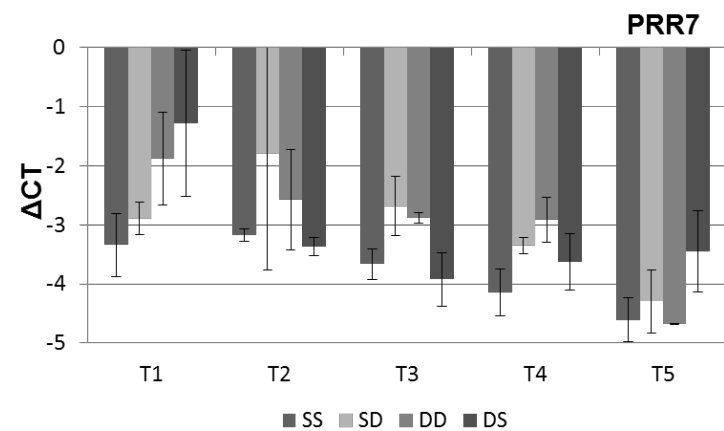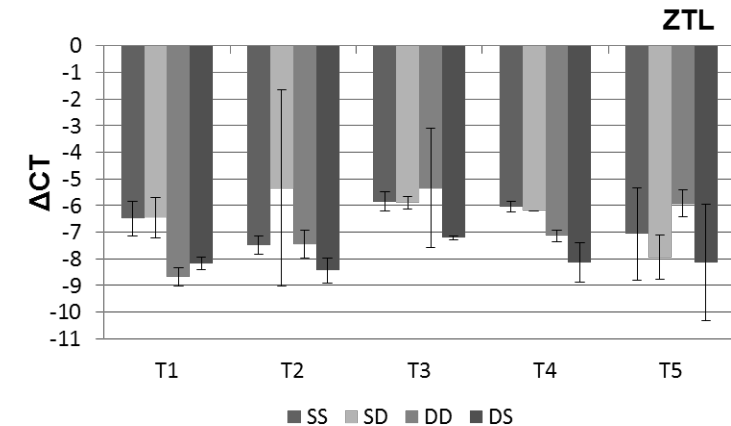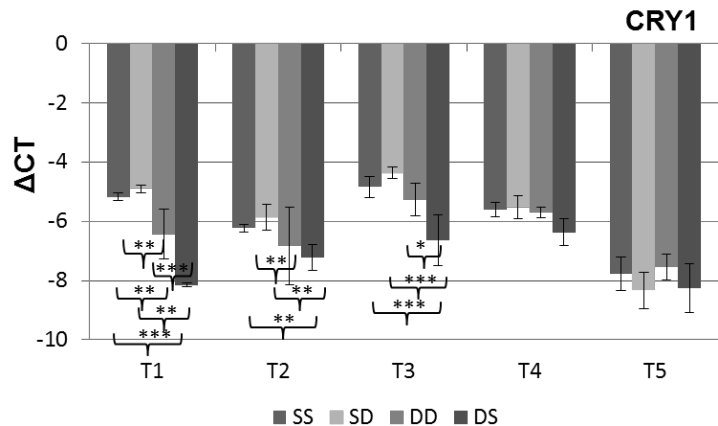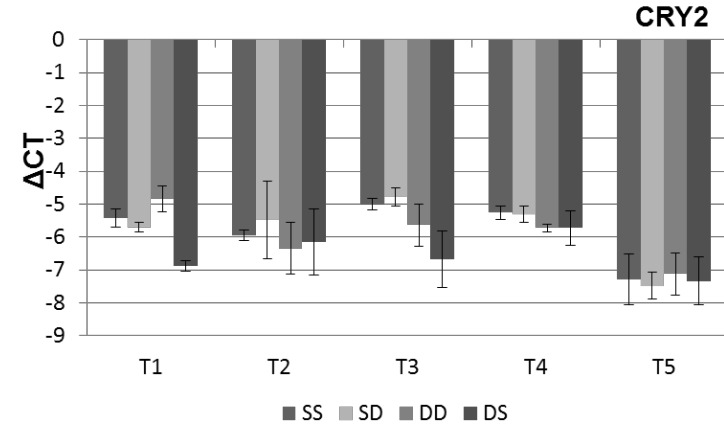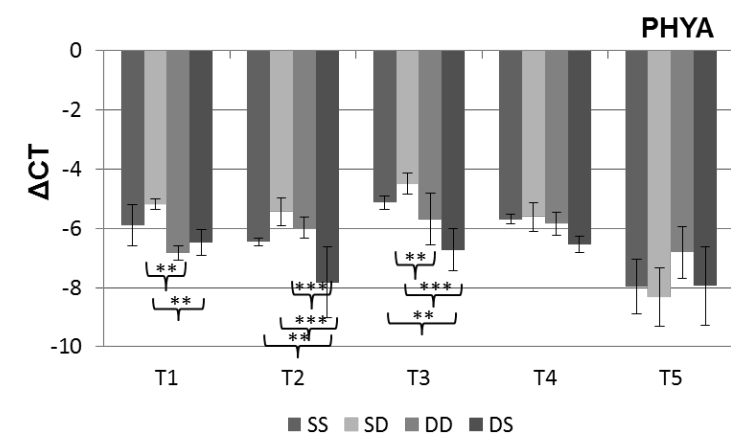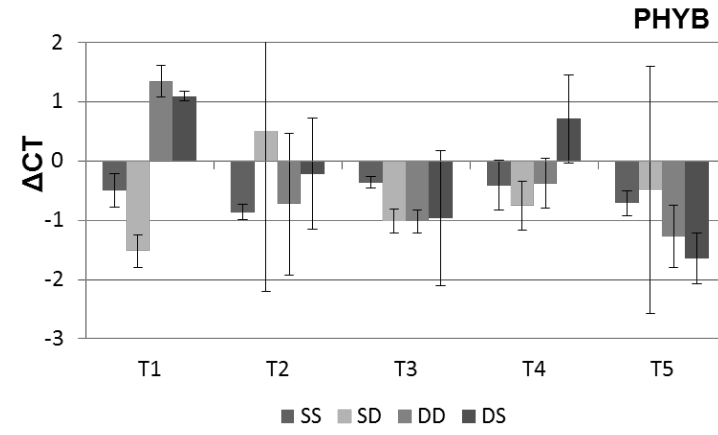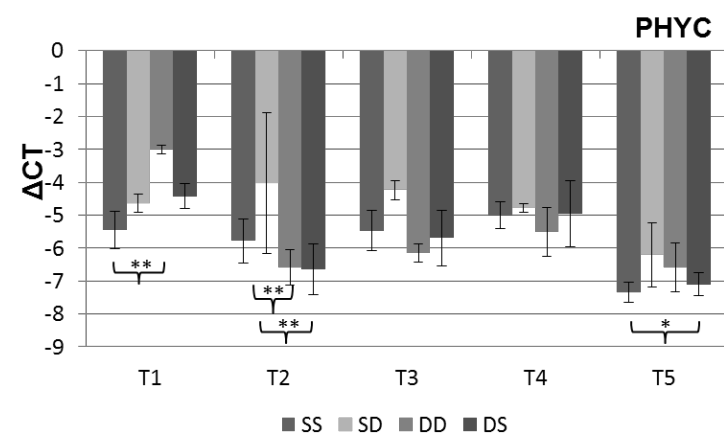

Supplement: Supplementary file 4 [file ECE3-7-1148-s004.pdf]

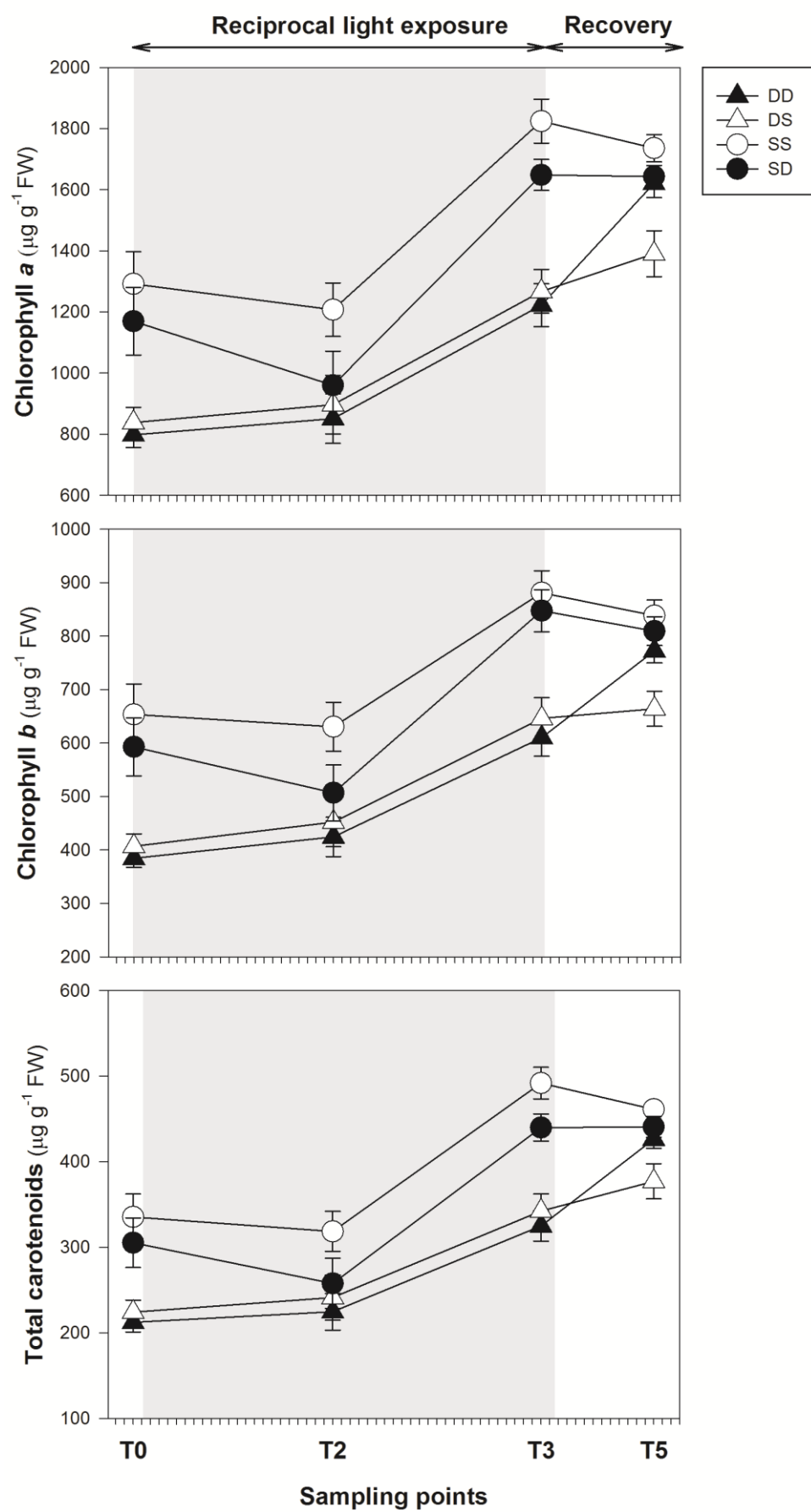

Supplement: Supplementary file 6 [file ECE3-7-1148-s006.pdf]

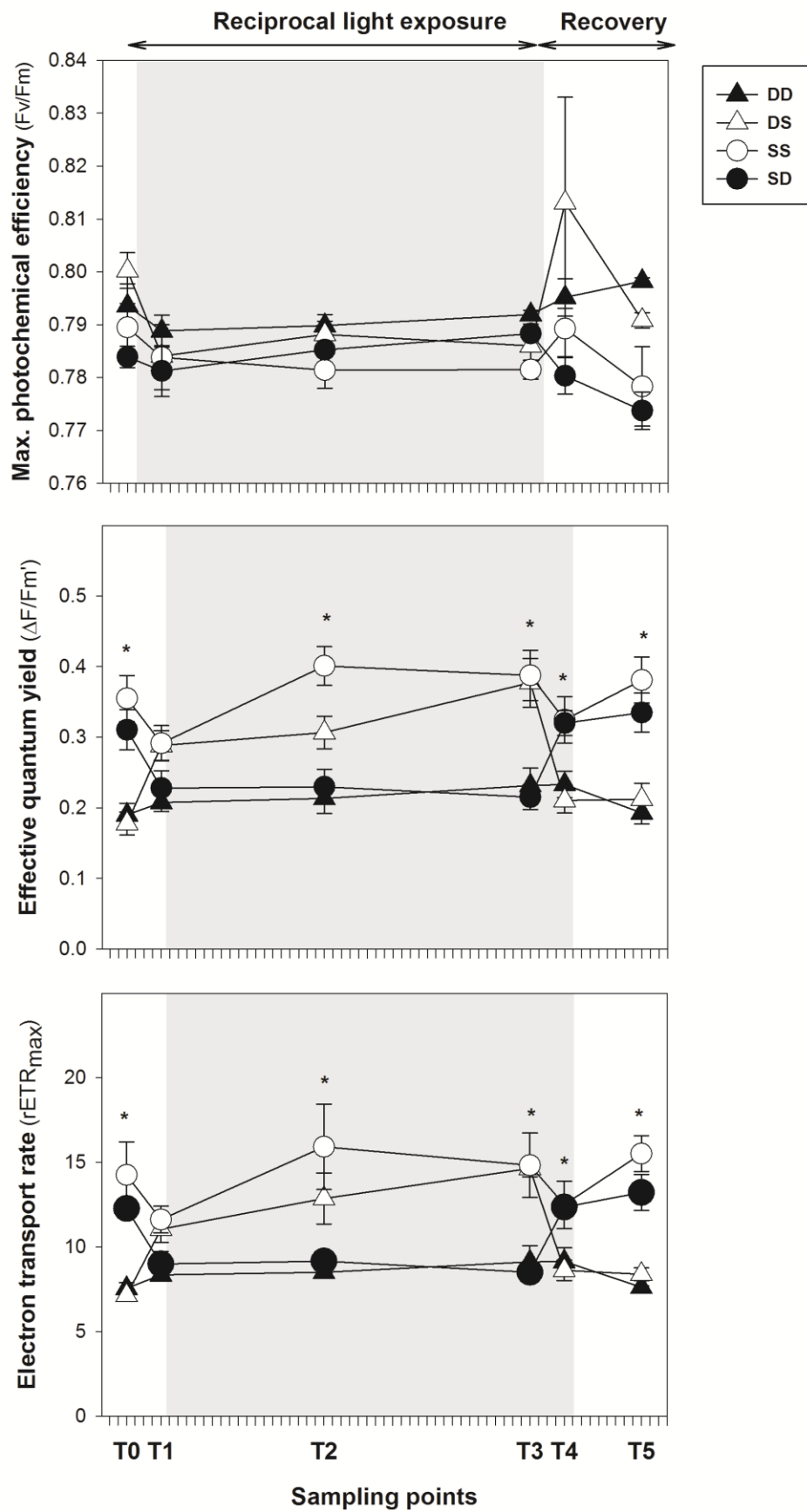

Supplement: Supplementary file 7 [file ECE3-7-1148-s007.pdf]
