# Supplementary material for: Long‐term acclimation to reciprocal light conditions suggests depth‐related selection in the marine foundation species Posidonia oceanica
Source: Ecol Evol. 2017 Jan 24;7(4):1148–64. doi: 10.1002/ece3.2731 (PMC5306012; doi:10.1002/ece3.2731)
Supplement: Supplementary file 5 [file ECE3-7-1148-s005.pdf]

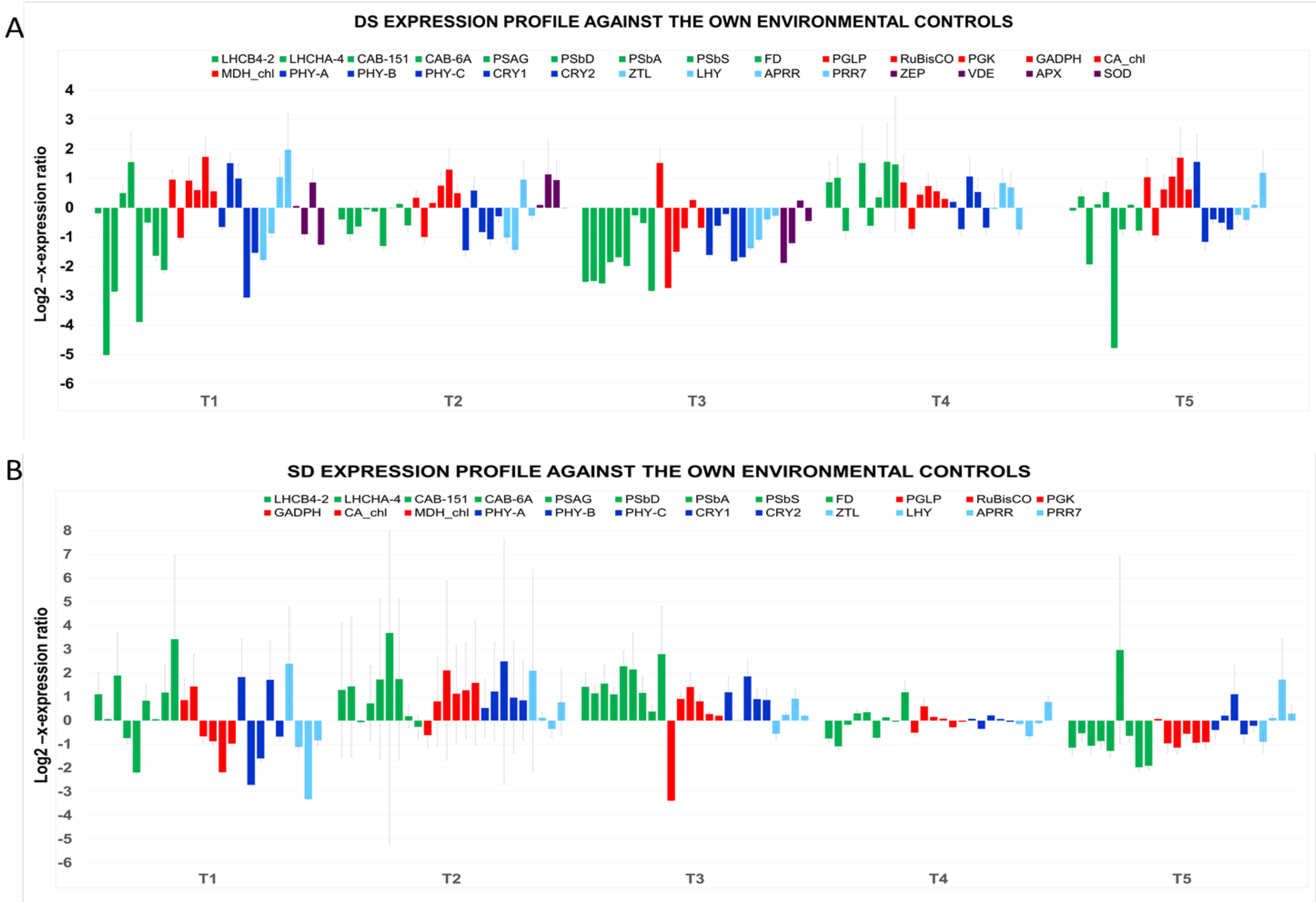
A
DS EXPRESSION PROFILE AGAINST THE OWN ENVIRONMENTAL CONTROLS
LHCB4-2 LHCHA-4 CAB-151 CAB-6A PSAG PSbD PSbA PSbS FD PGLP RuBisCO PGK GADPH CA_chl
MDH_chl PHY-A PHY-B PHY-C CRY1 CRY2 ZTL LHY APRR PRR7 ZEP VDE APX SOD
Log2 –x-expression ratio
4
3
2
1
0
-1
-2
-3
-4
-5
-6
T1
T2
T3
T4
T5
B
SD EXPRESSION PROFILE AGAINST THE OWN ENVIRONMENTAL CONTROLS
LHCB4-2 LHCHA-4 CAB-151 CAB-6A PSAG PSbD PSbA PSbS FD PGLP RuBisCO PGK
GADPH CA_chl MDH_chl PHY-A PHY-B PHY-C CRY1 CRY2 ZTL LHY APRR PRR7
Log2 –x-expression ratio
8
7
6
5
4
3
2
1
0
-1
-2
-3
-4
-5
-6
T1
T2
T3
T4
T5
