# Supplementary material for: Long‐term acclimation to reciprocal light conditions suggests depth‐related selection in the marine foundation species Posidonia oceanica
Source: Ecol Evol. 2017 Jan 24;7(4):1148–64. doi: 10.1002/ece3.2731 (PMC5306012; doi:10.1002/ece3.2731)
Supplement: Supplementary file 8 [file ECE3-7-1148-s008.docx]

**Table S1** List of genes selected for RT-qPCR experiments. List of genes selected for RT-qPCR experiments. Gene and protein names, biological process, and references are shown. TGR and REF indicate respectively, targets and references genes. For drimers specifically selected for this work .GenBank Accession Number, primer sequences, percent efficiency (*E*), and correlation coefficient (R^2^) are shown.

| **Pathways** | **Gene name** | **Symbol** | **references** | **TGR/**  **REF** | **Primers designed for this study** | | | | |
| --- | --- | --- | --- | --- | --- | --- | --- | --- | --- |
|  |  |  |  |  | **Primer Sequence 5’-3’** | | **GenBank** | **E** | **R2** |
| **Photoperiod** |  |  |  |  |  |  |  |  |  |
|  | Pseudo response regulators | **APRR** | this study | TGR | F:TAGATTGGTCACCGGAGCTT | R:CCTTCCTTCTTCGTCTTTCG | gb\|GEMD01055101.1 | 100% | 0.99 |
|  | Pseudo response regulators | **PRR7** | this study | TGR | F:CCGCTGCTCTGCTAGTCTCT | R:AATGGCAGTGGAAGTGGAAG | gb\|GEMD01003003.1 | 100% | 0.99 |
|  | Late Elongated Hypocotyl | **LHY** | this study | TGR | F: GCATCTTGGAAAAGGGTCAA | R: CGAAAGGCTTGTATGGGAAC | gb\|GEMD01030913.1 | 100% | 0.99 |
|  | Zeitlupe | **ZTL** | this study | TGR | F:CATCAAGGCACCTCCGTATT | R:TTACGTCAGCAAGTCGTTCG | gb\|GEMD01017027.1 | 100% | 0.99 |
|  | Gigantea | **GI** | this study | REF | F:GATTGGTCTGCTGTGCAAGA | R:AATGCTTGTTGATGGGGAAG | gb\|GEMD01021005.1 | 97% | 0.99 |
| **Photoreception** |  |  |  |  |  |  |  |  |  |
|  | Cryptochrome 1 | **CRY1** | this study | TGR | F:CTTTTGCAGCCTTCTGGAAC | R:AAGTGCATTGCTTGCCTTTT | gb\|GEMD01069944.1 | 100% | 0.99 |
|  | Cryptochrome 2 | **CRY2** | this study | TGR | F:TCCACGTGCAGAAGAAAGTG | R:CCTGCCTCCAAATCTTGAAA | gb\|GEMD01039801.1 | 100% | 0.98 |
|  | Phytochrome A | **PHY-A** | this study | TGR | F:TTGCGATTGATGAGAAGACG | R:ACTTCACCAAAACCCAGTGC | gb\|GEMD01012410.1 | 100% | 0.98 |
|  | Phytochrome B | **PHY-B** | this study | TGR | F:TCGGTGACCAAGTATGTGGA | R:GGATGGCATTCATTTCTGCT | gb\|GEMD01045656.1 | 90% | 0.99 |
|  | Phytochrome C | **PHY-C** | this study | TGR | F:CAGCCTGGGTTTTGAGGATA | R:TGGTTGTTTGCCATCACACT | gb\|GEMD01057042.1 | 96% | 0.99 |
| **Photosynthesis** | Chlorophyll a-b binding protein 4 | **LHCHA.4** | Mazzuca et al.2013 | TGR |  |  |  |  |  |
|  |  |  | Dattolo et al. 2014 |  |  |  |  |  |  |
|  | Chlorophyll a-b binding protein CP29.2 | **LHCB4.2** | Mazzuca et al. 2013 | TGR |  |  |  |  |  |
|  |  |  | Dattolo et al. 2014 |  |  |  |  |  |  |
|  | Chlorophyll a-b binding protein 151 | **CAB-151** | Mazzuca et al. 2013 | TGR |  |  |  |  |  |
|  |  |  | Dattolo et al. 2014 |  |  |  |  |  |  |
|  | Chlorophyll a-b binding protein 6A | **CAB.6A** | Mazzuca et al. 2013 | TGR |  |  |  |  |  |
|  |  |  | Dattolo et al. 2014 |  |  |  |  |  |  |
|  | Photosystem II protein D1 | **psbA** | Mazzuca et al. 2013 | TGR |  |  |  |  |  |
|  |  |  | Dattolo et al. 2014 |  |  |  |  |  |  |
|  | Photosystem I reaction center subunit V | **PSAG** | Mazzuca et al. 2013 | TGR |  |  |  |  |  |
|  |  |  | Dattolo et al. 2014 |  |  |  |  |  |  |
|  | Photosystem II  protein D2 | **psbD** | Mazzuca et al. 2013 | TGR |  |  |  |  |  |
|  |  |  | Dattolo et al. 2014 |  |  |  |  |  |  |
|  | Photosystem II  22 kDa protein | **PSBS** | Mazzuca et al. 2013 | TGR |  |  |  |  |  |
|  |  |  | Dattolo et al. 2014 |  |  |  |  |  |  |
|  | Ferredoxin | **FD** | Mazzuca et al. 2013 | TGR |  |  |  |  |  |
|  |  |  | Dattolo et al. 2014 |  |  |  |  |  |  |
| **Carbon metabolism** |  |  |  |  |  |  |  |  |  |
|  | RuBisCO  (SSU5B small subunit) | **RbcS** | Mazzuca et al. 2013 | TGR |  |  |  |  |  |
|  |  |  | Dattolo et al. 2014 |  |  |  |  |  |  |
|  | Phosphoglycerate kinase | **PGK** | this study | TGR | F:CCAATTGTTGTTGGGAAACC | R:AGGATGGATGTCGTTGGAAG | gb\|GEMD01032216.1 | 100% | 0.99 |
|  | Phosphoglycolate phosphatase | **PGLP** | this study | TGR | F:TGGAGGTGCCAGCTTAGAGT | R:TGTTTCAGAGCAACGGTCAG | gb\|GEMD01002107.1 | 97% | 0.99 |
|  | Carbonic anhydrase, chl | **CA** | this study | TGR | F:GAAAGGGAGGCTGTGAACCT | R:CAAAGTCGAAGTGTCCACCA | gb\|GEMD01028645.1 | 90% | 0.99 |
|  | Glyceraldehyde-3-phosphate dehydrogenase | **GADPH** | Serra et al., 2012 | TGR |  |  |  |  |  |
|  | Malate dehydrogenase, chl | **MDH** | this study | TGR | F:GGAATGGAACGTGCAGATTT | R:CTGGAATATTTGGCGCATTT | gb\|GEMD01020102.1 | 90% | 0.99 |
| **Photoprotection** |  |  |  |  |  |  |  |  |  |
|  | Zeaxanthin epoxidase | **ZEP** | Mazzuca et al. 2013 | TGR |  |  |  |  |  |
|  |  |  | Dattolo et al. 2014 |  |  |  |  |  |  |
|  | Violaxanthin de epoxidase | **VDE** | this study | TGR | F:GGAGTCTGTATCCGCCATGT | R:GAACTTGAAAGGGCATGGAA | gb\|GEMD01039073.1 | 100% | 0.99 |
| **ROS-scavenging system** |  |  |  |  |  |  |  |  |  |
|  | Ascorbate peroxidase | **APX** | this study | TGR | F:AGTTACCAAGAAGGGATGTCAA | R:GAGAGTTATCGGACGCCATGA | gb\|GEMD01020238.1 | 100% | 0.98 |
|  | Superoxide dismutase | **SOD** | Lauritano et al. 2015 | TGR |  |  |  |  |  |
| **Ubiquitin system** |  |  |  |  |  |  |  |  |  |
|  | NTUBC2 Ubiquitin-conjugating enzyme | **NTUBC** | Serra et al. 2012 | REF |  |  |  |  |  |
