# Supplementary material for: Long‐term acclimation to reciprocal light conditions suggests depth‐related selection in the marine foundation species Posidonia oceanica
Source: Ecol Evol. 2017 Jan 24;7(4):1148–64. doi: 10.1002/ece3.2731 (PMC5306012; doi:10.1002/ece3.2731)
Supplement: Supplementary file 9 [file ECE3-7-1148-s009.docx]

**Table S2** Genetic diversity. a) Sample Size, N° Alleles (mean of), No. Effective Alleles, Observed Heterozygosity, Expected Heterozygosity, and and percentage of Polymorphic Loci calculated for the four experimental groups (SS, SD, DS DD). b) Results of hierarchical analysis of molecular variance (AMOVA) conducted on SSR profiles of 48 individuals. 12% of differentiation was associated at the partitioning of samples in the two groups "shallow" and "deep" di. c) F-Statistics values associated at the partitioning of samples in the two groups "Shallow" and "Deep".

**a)**

| **Group** | **N** | **Na** | **Ne** | **I** | **Ho** | **He** |
| --- | --- | --- | --- | --- | --- | --- |
| **DD** | 11,966 | 1,897 | 1,542 | 0,429 | 0,420 | 0,280 |
| **DS** | 11,931 | 2,241 | 1,701 | 0,534 | 0,416 | 0,329 |
| **SD** | 11,828 | 3,034 | 2,085 | 0,710 | 0,482 | 0,396 |
| **SS** | 11,966 | 2,759 | 1,905 | 0,643 | 0,451 | 0,375 |

**b)**

| **Source** | **df** | **SS** | **MS** | **Est. Var.** | **%** |
| --- | --- | --- | --- | --- | --- |
| **Among Pops** | 1 | 46,010 | 46,010 | 0,871 | 12% |
| **Among Indiv** | 46 | 193,167 | 4,199 | 0,000 | 0% |
| **Within Indiv** | 48 | 306,500 | 6,385 | 6,385 | 88% |
| **Total** | 95 | 545,677 |  | 7,256 | 100% |

**c)**

| **F-Statistics** | **Value** | **P(rand >= data)** |
| --- | --- | --- |
| **Fst** | 0,141 | 0,001 |
| **Fis** | -0,207 | 1,000 |
| **Fit** | -0,036 | 0,795 |
