# Supplementary material for: Long‐term acclimation to reciprocal light conditions suggests depth‐related selection in the marine foundation species Posidonia oceanica
Source: Ecol Evol. 2017 Jan 24;7(4):1148–64. doi: 10.1002/ece3.2731 (PMC5306012; doi:10.1002/ece3.2731)
Supplement: Supplementary file 10 [file ECE3-7-1148-s010.docx]

**Table S3a,b,c,d** RT-qPCR expression values of reciprocal pairwise comparison. Relative Expression value of test groups against control groups measured at each time point of reciprocal light exposure and recovery. *Home vs Away* comparisons: **a)** Expression value of SD against SS. **c)** Expression value of DS against DD. *Local vs foreign* comparisons: **b)** Expression value of SD against DD. **d)** Expression value of DS against SS.

For each pairwise comparison, relative expression values (log2) and St.Dev obtained with REST 2002 (Pfaffl et al., 2002) were reported. Reciprocal comparisons statistical significant are indicated in bold

**Table S3** a)

| **SD vs SS** | **T1** |  | **T2** |  | **T3** |  | **T4** |  | **T5** |  |
| --- | --- | --- | --- | --- | --- | --- | --- | --- | --- | --- |
|  | **Log2FC** | **St.Dev** | **Log2FC** | **St.Dev** | **Log2FC** | **St.Dev** | **Log2FC** | **St.Dev** | **Log2FC** | **St.Dev** |
| **LHCB4-2** | -1,787 | 0,157 | -0,940 | 0,546 | -1,032 | 0,114 | -0,712 | 0,170 | -1,010 | 0,368 |
| **LHCHA-4** | **-2,432** | 0,069 | -0,994 | 0,504 | -1,443 | 0,195 | -1,043 | 0,127 | -0,400 | 0,330 |
| **CAB-151** | -0,717 | 0,272 | -1,279 | 0,629 | -0,456 | 0,115 | -0,217 | 0,099 | -1,364 | 0,382 |
| **CAB-6A** | -0,568 | 0,255 | 0,013 | 0,028 | -0,225 | 0,081 | 0,362 | 0,214 | -0,940 | 0,385 |
| **PSAG** | -1,702 | 0,160 | -0,378 | 0,408 | -1,007 | 0,085 | 0,395 | 0,170 | -1,147 | 0,322 |
| **psbD** | 0,221 | 0,217 | 1,336 | 2,689 | -0,027 | 0,012 | -0,683 | 0,097 | 3,104 | 4,596 |
| **PSBS** | -1,069 | 0,333 | **-2,359** | 0,231 | -1,178 | 0,107 | 0,000 | 0,000 | -1,838 | 0,201 |
| **psbA** | 0,609 | 0,618 | **1,996** | 4,245 | 0,967 | 0,606 | 0,170 | 0,073 | -0,761 | 0,355 |
| **FD** | -0,709 | 0,369 | -1,654 | 0,431 | -0,571 | 0,093 | 1,236 | 0,528 | -1,773 | 0,219 |
| **PGLP** | -0,187 | 0,137 | -0,541 | 0,474 | -1,146 | 0,116 | -0,538 | 0,175 | 0,137 | 0,182 |
| **RbcS** | -0,142 | 0,119 | -0,479 | 0,555 | 0,256 | 0,082 | 0,643 | 0,359 | -0,829 | 0,432 |
| **GADPH** | -0,880 | 0,243 | 0,254 | 0,484 | -0,413 | 0,074 | 0,095 | 0,041 | -0,725 | 0,346 |
| **PGK** | -0,714 | 0,307 | 1,042 | 1,865 | -0,011 | 0,004 | 0,194 | 0,091 | -1,010 | 0,321 |
| **CA** | -0,531 | 0,233 | 1,407 | 2,396 | -0,375 | 0,065 | -0,416 | 0,116 | -1,413 | 0,239 |
| **MDH** | -0,442 | 0,256 | 0,740 | 1,419 | -0,599 | 0,079 | -0,121 | 0,041 | -1,549 | 0,267 |
| **PHY-A** | 0,621 | 0,574 | 1,357 | 3,013 | 0,659 | 0,221 | 0,119 | 0,052 | -0,264 | 0,326 |
| **PHY-B** | -1,311 | 0,201 | 1,199 | 2,214 | -1,100 | 0,106 | -0,603 | 0,101 | 0,062 | 0,072 |
| **PHY-C** | 0,860 | 0,879 | **2,049** | 3,917 | 1,361 | 0,663 | 0,262 | 0,095 | 1,294 | 1,689 |
| **CRY1** | 0,159 | 0,142 | 0,725 | 1,578 | 0,499 | 0,167 | 0,107 | 0,046 | -0,451 | 0,389 |
| **CRY2** | -0,393 | 0,203 | 0,812 | 1,639 | 0,262 | 0,108 | -0,007 | 0,002 | -0,089 | 0,124 |
| **ZTL** | -0,079 | 0,077 | **2,510** | 4,612 | -0,025 | 0,010 | -0,103 | 0,035 | -0,762 | 0,485 |
| **LHY** | -1,072 | 0,253 | -0,360 | 0,408 | -1,805 | 0,050 | -0,620 | 0,128 | 0,244 | 0,332 |
| **APRR** | -0,648 | 0,290 | 1,179 | 2,088 | 0,542 | 0,270 | -0,068 | 0,034 | 1,860 | 2,191 |
| **PRR7** | 0,338 | 0,282 | 1,732 | 3,183 | 1,006 | 0,332 | 0,824 | 0,276 | 0,421 | 0,570 |

**Table S3** b)

| **SD vsDD** | **T1** |  | **T2** |  | **T3** |  | **T4** |  | **T5** |  |
| --- | --- | --- | --- | --- | --- | --- | --- | --- | --- | --- |
|  | **Log2FC** | **St.Dev** | **Log2FC** | **St.Dev** | **Log2FC** | **St.Dev** | **Log2FC** | **St.Dev** | **Log2FC** | **St.Dev** |
| **LHCB4-2** | -0,047 | 0,056 | 1,354 | 3,347 | 1,421 | 0,670 | 0,564 | 0,367 | 0,143 | 0,212 |
| **LHCHA-4** | -1,096 | 0,302 | 1,505 | 3,523 | 1,148 | 0,427 | 0,600 | 0,420 | 0,252 | 0,365 |
| **CAB-151** | **2,727** | 4,540 | -0,118 | 0,342 | 1,908 | 1,258 | 0,668 | 0,596 | -1,136 | 0,423 |
| **CAB-6A** | -1,185 | 0,331 | 0,838 | 2,179 | 1,239 | 0,447 | 0,450 | 0,302 | -0,106 | 0,138 |
| **PSAG** | **-3,346** | 0,037 | 1,798 | 4,032 | **2,289** | 0,777 | 1,235 | 0,768 | -0,080 | 0,098 |
| **psbD** | -0,318 | 0,251 | **3,758** | 9,990 | **2,153** | 1,603 | 1,887 | 1,265 | -0,765 | 0,380 |
| **PSBS** | 0,023 | 0,031 | 0,250 | 0,709 | 0,376 | 0,277 | 2,421 | 1,726 | 0,810 | 1,148 |
| **psbA** | 0,051 | 0,072 | **2,073** | 4,930 | 1,357 | 1,004 | 0,914 | 0,537 | -0,743 | 0,367 |
| **FD** | **2,274** | 3,306 | -0,202 | 0,439 | **2,804** | 2,069 | 4,613 | 6,512 | -0,032 | 0,046 |
| **PGLP** | 0,417 | 0,695 | -0,647 | 0,617 | -0,865 | 0,527 | -0,762 | 0,334 | 0,794 | 1,041 |
| **RbcS** | 0,281 | 0,382 | 0,870 | 2,222 | 0,911 | 0,343 | 0,599 | 0,381 | -0,519 | 0,414 |
| **GADPH** | -0,737 | 0,300 | 1,394 | 3,129 | 0,994 | 0,413 | 0,919 | 0,483 | 0,979 | 1,282 |
| **PGK** | -1,821 | 0,235 | **2,178** | 4,594 | 1,415 | 0,638 | 0,865 | 0,515 | 0,847 | 1,091 |
| **CA** | -1,833 | 0,235 | 1,696 | 3,391 | 0,359 | 0,128 | 0,236 | 0,140 | 1,329 | 1,572 |
| **MDH** | 0,356 | 0,443 | **2,252** | 4,801 | 0,274 | 0,121 | 0,606 | 0,338 | -0,248 | 0,258 |
| **PHY-A** | 0,676 | 0,860 | 0,598 | 1,512 | 1,191 | 0,693 | 0,077 | 0,057 | -1,293 | 0,382 |
| **PHY-B** | -1,961 | 0,160 | 1,788 | 4,001 | -0,014 | 0,007 | -0,140 | 0,075 | 0,642 | 0,747 |
| **PHY-C** | **-2,126** | 0,137 | **2,861** | 7,103 | **2,108** | 0,914 | 0,765 | 0,643 | 0,491 | 0,659 |
| **CRY1** | 0,564 | 0,818 | 1,033 | 2,769 | 0,903 | 0,439 | 0,038 | 0,025 | -0,560 | 0,412 |
| **CRY2** | -1,832 | 0,168 | 0,920 | 2,091 | 0,867 | 0,513 | 0,262 | 0,153 | -0,136 | 0,181 |
| **ZTL** | 0,234 | 0,349 | **2,166** | 5,014 | -0,558 | 0,254 | 0,797 | 0,468 | -1,789 | 0,247 |
| **LHY** | **-2,268** | 0,124 | 0,182 | 0,391 | 0,240 | 0,115 | 0,597 | 0,343 | -0,014 | 0,020 |
| **APRR** | **-4,466** | 0,012 | -0,293 | 0,384 | 0,922 | 0,413 | -0,494 | 0,169 | 1,709 | 2,373 |
| **PRR7** | -1,992 | 0,150 | 0,835 | 1,785 | 0,201 | 0,073 | -0,592 | 0,140 | 0,604 | 0,822 |

**Table S3** c)

| **DS vsDD** | **T1** |  | **T2** |  | **T3** |  | **T4** |  | **T5** |  |
| --- | --- | --- | --- | --- | --- | --- | --- | --- | --- | --- |
|  | **Log2FC** | **St.Dev** | **Log2FC** | **St.Dev** | **Log2FC** | **St.Dev** | **Log2FC** | **St.Dev** | **Log2FC** | **St.Dev** |
| **LHCB4-2** | **2,100** | 2,217 | **2,395** | 3,858 | 0,029 | 0,017 | 1,053 | 0,905 | 0,098 | 0,078 |
| **LHCHA-4** | **-3,134** | 0,087 | **2,101** | 3,708 | 0,192 | 0,126 | 1,206 | 1,006 | 0,581 | 0,451 |
| **CAB-151** | -0,283 | 0,287 | 0,212 | 0,474 | -0,845 | 0,230 | -1,012 | 0,338 | -2,456 | 0,089 |
| **CAB-6A** | 0,118 | 0,148 | 0,911 | 1,589 | -0,601 | 0,151 | 0,061 | 0,053 | 0,194 | 0,154 |
| **PSAG** | 0,458 | 0,413 | 2,541 | 4,001 | 1,707 | 0,587 | 1,705 | 1,568 | 0,727 | 0,584 |
| **psbD** | **-3,880** | 0,018 | 1,618 | 3,289 | 0,292 | 0,228 | -0,432 | 0,244 | -4,585 | 0,012 |
| **PSBS** | 0,004 | 0,004 | **3,238** | 5,330 | 1,133 | 0,805 | 1,749 | 1,598 | 0,298 | 0,227 |
| **psbA** | -1,150 | 0,266 | 0,068 | 0,101 | 0,080 | 0,065 | 0,437 | 0,375 | -0,863 | 0,218 |
| **FD** | 1,409 | 1,599 | 1,349 | 2,234 | 0,639 | 0,481 | 1,658 | 2,662 | -0,581 | 0,213 |
| **PGLP** | 1,913 | 2,723 | 0,506 | 0,876 | **2,002** | 3,991 | 1,041 | 1,263 | 1,230 | 0,921 |
| **RbcS** | -0,052 | 0,050 | 0,852 | 1,379 | -1,983 | 0,074 | -0,537 | 0,229 | -0,749 | 0,232 |
| **GADPH** | 0,733 | 0,637 | 1,934 | 2,916 | 0,507 | 0,199 | 0,889 | 0,654 | 1,257 | 0,990 |
| **PGK** | 0,367 | 0,525 | 1,797 | 2,542 | 0,027 | 0,011 | 0,630 | 0,467 | 0,817 | 0,713 |
| **CA** | 0,533 | 0,782 | 1,579 | 2,124 | 0,985 | 0,372 | 0,621 | 0,453 | 2,138 | 1,610 |
| **MDH** | 0,787 | 0,755 | 1,484 | 2,072 | -0,298 | 0,097 | 0,206 | 0,164 | 0,615 | 0,466 |
| **PHY-A** | -0,051 | 0,047 | -1,709 | 0,282 | -0,976 | 0,189 | -0,546 | 0,237 | -0,965 | 0,341 |
| **PHY-B** | 0,643 | 0,557 | 0,517 | 1,002 | -0,083 | 0,051 | 1,346 | 1,182 | -1,004 | 0,229 |
| **PHY-C** | -1,711 | 0,144 | -0,006 | 0,011 | 0,524 | 0,241 | 0,674 | 0,686 | -0,533 | 0,274 |
| **CRY1** | **-2,107** | 0,136 | -0,265 | 0,355 | -1,321 | 0,146 | -0,499 | 0,230 | -0,549 | 0,291 |
| **CRY2** | **-2,424** | 0,074 | 0,315 | 0,515 | -0,983 | 0,174 | 0,145 | 0,115 | -0,052 | 0,045 |
| **ZTL** | -0,920 | 0,287 | -0,861 | 0,448 | -1,816 | 0,144 | -0,840 | 0,250 | -2,044 | 0,191 |
| **LHY** | -1,523 | 0,170 | -0,397 | 0,331 | 1,055 | 0,500 | 1,027 | 0,737 | -0,216 | 0,154 |
| **APRR** | **-2,227** | 0,106 | -0,013 | 0,017 | 0,081 | 0,047 | 0,877 | 0,739 | 0,296 | 0,433 |
| **PRR7** | 0,191 | 0,249 | -0,669 | 0,401 | -0,976 | 0,096 | -0,559 | 0,206 | 1,391 | 1,053 |

**Table S3** d)

| **DS vsSS** | **T1** |  | **T2** |  | **T3** |  | **T4** |  | **T5** |  |
| --- | --- | --- | --- | --- | --- | --- | --- | --- | --- | --- |
|  | **Log2 FC** | **St.Dev** | **Log2FC** | **St.Dev** | **Log2FC** | **St.Dev** | **Log2FC** | **St.Dev** | **Log2FC** | **St.Dev** |
| **LHCB4-2** | 0,359 | 0,275 | 0,101 | 0,104 | **-2,425** | 0,048 | -0,223 | 0,139 | -1,055 | 0,192 |
| **LHCHA-4** | **-4,471** | 0,017 | -0,398 | 0,297 | **-2,400** | 0,098 | -0,437 | 0,164 | -0,071 | 0,049 |
| **CAB-151** | **-3,727** | 0,016 | -0,949 | 0,342 | **-3,209** | 0,028 | -1,898 | 0,163 | -2,685 | 0,077 |
| **CAB-6A** | 0,735 | 0,492 | 0,086 | 0,094 | **-2,064** | 0,079 | -0,026 | 0,020 | -0,640 | 0,207 |
| **PSAG** | **2,103** | 1,428 | 0,365 | 0,322 | -1,589 | 0,060 | 0,865 | 0,695 | -0,339 | 0,174 |
| **psbD** | **-3,342** | 0,023 | -0,804 | 0,278 | -1,889 | 0,072 | -3,002 | 0,040 | -0,716 | 0,427 |
| **PSBS** | -1,089 | 0,269 | 0,629 | 0,716 | -0,422 | 0,099 | -0,672 | 0,224 | -2,350 | 0,065 |
| **psbA** | -0,593 | 0,160 | -0,009 | 0,009 | -0,310 | 0,142 | -0,307 | 0,152 | -0,880 | 0,197 |
| **FD** | -1,575 | 0,187 | -0,103 | 0,095 | **-2,737** | 0,024 | -1,719 | 0,139 | -2,322 | 0,066 |
| **PGLP** | 1,308 | 0,500 | 0,612 | 0,606 | 1,721 | 0,630 | 1,265 | 0,756 | 0,574 | 0,440 |
| **RbcS** | -0,476 | 0,128 | -0,497 | 0,268 | **-2,639** | 0,038 | -0,493 | 0,210 | -1,059 | 0,215 |
| **GADPH** | 0,590 | 0,349 | 0,794 | 0,740 | -0,900 | 0,074 | 0,065 | 0,044 | -0,448 | 0,188 |
| **PGK** | 1,473 | 1,317 | 0,661 | 0,574 | -1,400 | 0,072 | -0,041 | 0,025 | -1,040 | 0,214 |
| **CA** | 1,834 | 1,086 | 1,290 | 1,088 | 0,250 | 0,079 | -0,031 | 0,019 | -0,604 | 0,203 |
| **MDH** | -0,011 | 0,008 | -0,028 | 0,028 | -1,172 | 0,085 | -0,521 | 0,177 | -0,687 | 0,207 |
| **PHY-A** | -0,107 | 0,041 | -0,950 | 0,328 | -1,509 | 0,108 | -0,504 | 0,176 | 0,064 | 0,087 |
| **PHY-B** | 1,293 | 0,411 | -0,071 | 0,095 | -1,170 | 0,147 | 0,883 | 0,621 | -1,584 | 0,160 |
| **PHY-C** | 1,275 | 0,755 | -0,818 | 0,261 | -0,224 | 0,084 | 0,171 | 0,116 | 0,270 | 0,279 |
| **CRY1** | -2,513 | 0,029 | -0,573 | 0,294 | -1,726 | 0,093 | -0,430 | 0,180 | -0,440 | 0,276 |
| **CRY2** | -0,986 | 0,116 | 0,207 | 0,261 | -1,589 | 0,096 | -0,124 | 0,068 | -0,005 | 0,004 |
| **ZTL** | -1,234 | 0,105 | -0,517 | 0,248 | -1,284 | 0,084 | -1,740 | 0,132 | -1,017 | 0,441 |
| **LHY** | -0,328 | 0,122 | -0,939 | 0,250 | -0,991 | 0,083 | -0,190 | 0,095 | 0,042 | 0,037 |
| **APRR** | 1,590 | 1,219 | 1,459 | 1,527 | -0,300 | 0,124 | 1,303 | 0,963 | 0,447 | 0,566 |
| **PRR7** | **2,520** | 2,501 | 0,228 | 0,240 | -0,172 | 0,047 | 0,857 | 0,583 | 1,208 | 0,893 |
| **ZEP** | 0,606 | 0,468 | 0,594 | 0,570 | -1,782 | 0,058 | -0,078 | 0,049 | -1,504 | 0,168 |
| **VDE** | -0,352 | 0,164 | 1,638 | 2,719 | -1,108 | 0,097 | -0,357 | 0,153 | -4,067 | 0,029 |
| **APX** | 1,411 | 1,003 | 1,441 | 1,428 | 0,344 | 0,098 | 0,897 | 0,560 | 0,125 | 0,087 |
| ***SOD*** | *-0,713* | *0,152* | *0,491* | *0,574* | *-0,355* | *0,086* | *1,208* | *0,767* | *-* | *-* |
