# Supplementary material for: Long‐term acclimation to reciprocal light conditions suggests depth‐related selection in the marine foundation species Posidonia oceanica
Source: Ecol Evol. 2017 Jan 24;7(4):1148–64. doi: 10.1002/ece3.2731 (PMC5306012; doi:10.1002/ece3.2731)
Supplement: Supplementary file 11 [file ECE3-7-1148-s011.docx]

**Table S4** ANOSIM –SIMPER. Pairwise comparisons of gene expression (assessed as -ΔCT) in the seagrass *P. oceanica* among groups at five time points during the reciprocal transplantation experiment using ANOSIM and SIMPER. Global R and significance values for single time points (T1–T5) and significance values for pair wise population comparisons taken form ANOSIM are shown. Contribution (higher than 5%) of single target genes to total gene expression differences among groups (assessed as -ΔCT -values), given for all four comparisons between tests groups and the corresponding controls (native and foreign) during the exposure and the recovery phase, after SIMPER analysis were also indicated.

| **PAIR-WISE COMPARISONS** | | **EXPOSURE** | | | **RECOVERY** | |
| --- | --- | --- | --- | --- | --- | --- |
|  |  | **T1** | **T2** | **T3** | **T4** | **T5** |
| **LOW LIGHT** | |  |  |  |  |  |
| ***Shallow*** |  |  |  |  |  |  |
|  | ***SD vs DD*** |  |  |  |  |  |
|  | ***Av. Diss*** | ***3.91*** | ***2.95*** | ***2.03*** | ***1.78*** | ***2.34*** |
|  | **Genes** | ZTL (12.30) |  | PGLP (16.38) | FD (14.76) | APRR (9.73) |
|  |  |  | psbD (5.91) | ZTL (5.58) | PGLP (11.67) | CA (5.88) |
|  |  |  | PHY-C (5.83) | FD ( 5.41) | PSBS (6.93) | PHY-B (5.55) |
|  |  | APRR (6.78) | ZTL (5.30) |  |  | PGLP (5.34) |
|  |  | CAB-151 (6.47) | CAB-151 (5.03) |  |  |  |
|  |  | FD (5.36) |  |  | psbD (5.04) |  |
|  | ***SD vs SS*** |  |  |  |  |  |
|  | ***Av. Diss*** | ***1.84*** | ***2.87*** | ***2.09*** | ***1.22*** | ***1.89*** |
|  | **Genes** | LHCA.4 (7.64) | PSBS (6.91) | LHY (7.29) |  | psbD (10.29) |
|  |  | PHY-B (7.36) | APRR (5.90) | PHY-B (7.27) | PGLP (7.68) |  |
|  |  | LHCB4.2 (5.98) | FD (5.77) | PGLP (7.19) | APRR (6.49) | PHY-C (6.71) |
|  |  | PSAG (5.69) | ZTL (5.13) | LHCA-4 (6.30) | FD (5.94) |  |
|  |  |  | CAB-151 (5.09) | PSBS (5.21) |  |  |
| **HIGH LIGHT** | |  |  |  |  |  |
| ***Deep*** |  |  |  |  |  |  |
|  | ***DS vs SS*** |  |  |  |  |  |
|  | ***Av. Diss*** | ***2.88*** | ***1.69*** | ***2.25*** | ***1.6*** | ***2.27*** |
|  | **Genes** | LHCA.4 (9.03) | L23 (7.43) | PHY-B (6.68) | APRR (8.84) |  |
|  |  | CAB-151 (8.40) |  | PGLP (6.47) | psbD (8.66) | psbD (7.19) |
|  |  | psbD (7.02) | PHY-B (6.98) | CAB-151 (6.46) | PGLP (7.49) | CAB-151 (5.62) |
|  |  |  | PHY-C (5.98) | FD (5.47) | PHY-B (6.46) |  |
|  |  | CRY1 (6.41) | CAB-151 (5.58) | LHCA-4 (5.37) | CAB-151 (5.66) | ZTL (5.17) |
|  |  |  | APRR (5.05) |  | ZTL (5.24) |  |
|  |  |  | PHY-A (5.03) |  | PRR7 (5.01) |  |
|  | ***DS vs DD*** |  |  |  |  |  |
|  | ***Av. Diss*** | ***3.98*** | ***2.92*** | ***2.08*** | ***1.88*** | ***2.65*** |
|  | **Genes** | ZTL (12.02) | PSBS (6.42) | PHY-B (6.02) | PGLP (9.38) |  |
|  |  |  |  | ZTL (5.65) | FD (7.80) | psbD (7.87) |
|  |  |  |  | PSAG (5.41) | psbD (6.14) | CA (6.54) |
|  |  | PGLP (5.32) |  |  | PHY-B (6.06) |  |
|  |  |  |  |  | PSBS (5.79) | PGLP (5.30) |
|  |  |  |  |  | APRR (5.59) |  |
|  |  |  |  |  | PSAG (5.16) | PRR7 (5.03) |
| ***Controls*** |  |  |  |  |  |  |
|  | ***SS vs DD*** |  |  |  |  |  |
|  | ***Av. Diss*** | ***4.18*** | ***2.72*** | ***2.43*** | ***1.73*** | ***2.43*** |
|  | **Genes** | ZTL (11.35) |  | FD (7.51) | PGLP (11.34) | CA (7.66) |
|  |  |  | PSBS (6.16) | PGLP (7.29) | FD (10.97) | psbD (7.41) |
|  |  | CAB-151 (7.20) | psbD (5.80) | PsaG (7.03) | psbD (6.97) | PSBS (6.88) |
|  |  |  | LHCA-4 (5.59) | LHCA-4 (5.52) | PSBS (6.85) |  |
|  |  | FD (6.28) | LHCB-4.2 (5.28) |  | PRR7 (6.03) | FD (5.28) |
|  |  |  | PSAG (5.17) |  | APRR (5.97) |  |
|  |  |  |  |  |  |  |
|  |  |  |  |  |  |  |
| **Global R** | | **0.642** | **0.178** | **0.41** | **0.429** | **0.454** |
| **Global P** | | **0.001** | **0.011** | **0.009** | **0.002** | **0.005** |
