# Supplementary material for: Long‐term acclimation to reciprocal light conditions suggests depth‐related selection in the marine foundation species Posidonia oceanica
Source: Ecol Evol. 2017 Jan 24;7(4):1148–64. doi: 10.1002/ece3.2731 (PMC5306012; doi:10.1002/ece3.2731)
Supplement: Supplementary file 12 [file ECE3-7-1148-s012.docx]

**Table S5** Statistical analysis (ANOVA) of Pigments, photosynthetic and respiration rates. Results of the repeated measures ANOVA testing for the effects of growing under reciprocal light exposure of deep and shallow *P. oceanica* plants over time, and of the one-way ANOVA testing for the effects at the end of the recovery period for pigments (chlorophyll a, b and total carotenoids) and parameters derived from P-E curves (gross photosynthetic rates and respiratory rates).

| Reciprocal light exposure period^a^ | | | |  | Recovery period^b^ | | | |
| --- | --- | --- | --- | --- | --- | --- | --- | --- |
| Effect | df | F | p |  | Effect | df | F | p |
| **Pigments** |  |  |  |  |  |  |  |  |
| *Chlorophyll a (μg cm^-2^)* |  |  |  |  |  |  |  |  |
| Treatment | 3 | 9.635 | ** |  | Treatment | 3 | 4.253 | * |
| Time | 2 | 0.438 | n.s. |  |  |  |  |  |
| Tr x T | 6 | 0.323 | n.s. |  | *Post-hoc* | DS^a^ SD^ab^ DD^ab^ SS^b^ | | |
|  |  |  |  |  |  |  |  |  |
| *Chlorophyll b (μg cm^-2^)* |  |  |  |  |  |  |  |  |
| Treatment | 3 | 14.758 | ** |  | Treatment | 3 | 2.44 | n.s. |
| Time | 2 | 0.419 | n.s. |  |  |  |  |  |
| Tr x T | 6 | 0.351 | n.s. |  |  |  |  |  |
|  |  |  |  |  |  |  |  |  |
| *Total carotenoids (μg cm^-2^)* |  |  |  |  |  |  |  |  |
| Treatment | 3 | 9.025 | ** |  | Treatment | 3 | 2.415 | n.s. |
| Time | 2 | 0.103 | n.s. |  |  |  |  |  |
| Tr x T | 6 | 0.256 | n.s. |  |  |  |  |  |
|  |  |  |  |  |  |  |  |  |
| **P-E curves** |  |  |  |  |  |  |  |  |
| *Gross photosynthetic rate - grossP_max_ (μmol O_2_ g^-1^ h^-1^)* | | | |  |  |  |  |  |
| Treatment | 3 | 4.318 | * |  | Treatment | 3 | 25.30 | *** |
| Time | 2 | 36.129 | *** |  |  |  |  |  |
| Tr x T | 6 | 4.378 | * |  | *Post-hoc* | DS^a^ DD^b^ SS^b^ SD^c^ | | |
|  |  |  |  |  |  |  |  |  |
| *Respiratory rates (μmol O_2_ g^-1^ h^-1^)* | | | |  |  |  |  |  |
| Treatment | 3 | 3.375 | n.s. |  | Treatment | 3 | 10.97 | ** |
| Time | 2 | 9.663 | ** |  |  |  |  |  |
| Tr x T | 6 | 11.37 | *** |  | *Post-hoc* | DS^a^ DD^b^ SS^b^ SD^b^ | | |
|  |  |  |  |  |  |  |  |  |

**^a^** Repeated measures ANOVA results for the reciprocal light exposure period. Data were first checked for sphericity and when failed (p<0.05) the p value and degrees of freedom were adjusted accordingly. Modified p values are shown, though degrees of freedom are unmodified. ^b^ One-way Anova results for the end of the recovery period. Post-hoc: Tukey’s post-hoc analysis. Different letters indicate significant differences among treatments
