# Supplementary material for: Long‐term acclimation to reciprocal light conditions suggests depth‐related selection in the marine foundation species Posidonia oceanica
Source: Ecol Evol. 2017 Jan 24;7(4):1148–64. doi: 10.1002/ece3.2731 (PMC5306012; doi:10.1002/ece3.2731)
Supplement: Supplementary file 13 [file ECE3-7-1148-s013.docx]

**Table S6** Statistical analysis (ANOVA) of plants growth under reciprocal light exposure. Results of the repeated measures ANOVA testing for the differences in growth under reciprocal light exposure of deep and shallow *P. oceanica* plants over time, and of the one-way ANOVA testing for the effects at the end of the recovery period.

| Reciprocal light exposure period | | | |  | Recovery period | | |
| --- | --- | --- | --- | --- | --- | --- | --- |
| Effect | df | F | p |  | df | F | p |
|  |  |  |  |  |  |  |  |
| *Maximum quantum yield of PSII (Fv/Fm)* | | |  |  |  |  |  |
| Treatment | 3 | 1.76 | n.s. |  | 3 | 2.799 | n.s. |
| Time | 3 | 8.23 | *** |  | 2 | 2.925 | n.s. |
| Tr x T | 9 | 2.89 | * |  | 6 | 2.123 | n.s. |
|  |  |  |  |  |  |  |  |
| *Effective quantum yield (ΔF/Fm’)* | |  |  |  |  |  |  |
| Treatment | 3 | 29.55 | *** |  | 3 | 19.50 | *** |
| Time | 3 | 4.34 | * |  | 2 | 5.84 | *. |
| Tr x T | 9 | 7.73 | *** |  | 6 | 3.59 | * |
|  |  |  |  |  |  |  |  |
| *Relative electron transport rate (rETRmax)* | | |  |  |  |  |  |
| Treatment | 3 | 28.59 | *** |  | 3 | 25.61 | *** |
| Time | 3 | 4.42 | * |  | 2 | 1.12 | n.s. |
| Tr x T | 9 | 7.56 | *** |  | 6 | 9.37 | *** |
